# Disruption to the FOXO-PRDM1 axis resulting from deletions of chromosome 6 in acute lymphoblastic leukaemia

**DOI:** 10.1038/s41375-023-01816-0

**Published:** 2023-01-20

**Authors:** Paul B. Sinclair, Ruth E. Cranston, Prahlad Raninga, Joanna Cheng, Rebecca Hanna, Zoe Hawking, Steven Hair, Sarra L. Ryan, Amir Enshaei, Sirintra Nakjang, Vikki Rand, Helen J. Blair, Anthony V. Moorman, Olaf Heidenreich, Christine J. Harrison

**Affiliations:** 1 Newcastle University Centre for Cancer, Translational and Clinical Research Institute, Faculty of Medical Sciences, Newcastle University, Newcastle-Upon-Tyne, UK; 2 Bioinformatics Support Unit, Faculty of Medical Science, Newcastle University, Newcastle-Upon-Tyne, UK; 3 School of Health and Life Sciences, Teesside University, Middlesborough, UK; 4 National Horizons Centre, Teesside University, Darlington, UK; 5 Princess Maxima Centre for Paediatric Oncology, Utrecht, The Netherlands

**Keywords:** Acute lymphocytic leukaemia, Cancer genomics, Oncogenes

## Abstract

A common problem in the study of human malignancy is the elucidation of cancer driver mechanisms associated with recurrent deletion of regions containing multiple genes. Taking B-cell acute lymphoblastic leukaemia (B-ALL) and large deletions of 6q [del(6q)] as a model, we integrated analysis of functional cDNA clone tracking assays with patient genomic and transcriptomic data, to identify the transcription factors FOXO3 and PRDM1 as candidate tumour suppressor genes (TSG). Analysis of cell cycle and transcriptomic changes following overexpression of *FOXO3* or *PRDM1* indicated that they co-operate to promote cell cycle exit at the pre-B cell stage. *FOXO1* abnormalities are absent in B-ALL, but like *FOXO3*, *FOXO1* expression suppressed growth of *TCF3::PBX1* and *ETV6::RUNX1* B-ALL in-vitro. While both FOXOs induced *PRDM1* and other genes contributing to late pre-B cell development, FOXO1 alone induced the key transcription factor, *IRF4*, and chemokine, *CXCR4*. CRISPR-Cas9 screening identified FOXO3 as a TSG, while FOXO1 emerged as essential for B-ALL growth. We relate this FOXO3-specific leukaemia-protective role to suppression of glycolysis based on integrated analysis of CRISPR-data and gene sets induced or suppressed by FOXO1 and FOXO3. Pan-FOXO agonist Selinexor induced the glycolysis inhibitor TXNIP and suppressed B-ALL growth at low dose (ID_50_ < 50 nM).

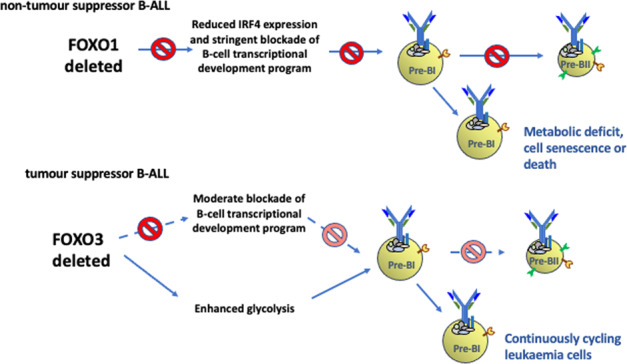

## Introduction

Among lymphoid malignancies, deletions or loss of heterozygosity of the long arm of chromosome 6 (6q) have been reported to occur in B- and T-ALL, chronic lymphocytic leukaemia (CLL) and various lymphomas at frequencies as high as 90% [1–5]. Hampering attempts to define common deleted regions (CDR) and critical target genes, these deletions are usually large, sometimes complex and invariably hemizygous. However, bona fide tumour suppressor activity has been demonstrated for *PRDM1* (*BLIMP1*) in diffuse large B-cell lymphomas, natural killer cell leukaemia/lymphoma and systemic anaplastic large cell lymphoma, where homozygous inactivation, through mutation unmasked by 6q deletion, has been demonstrated [6–8]. Likewise, *EPHA7* was shown to be silenced through combinations of deletion and promoter methylation in follicular lymphoma and T-cell lymphoma/leukaemia [9]. Other candidate tumour suppressor genes (TSG) in the region include *GRIK2*, implicated from an inherited mutation found in a case of childhood B-ALL carrying a 6q deletion [10], while loss of BACH2 and CCNC function have been associated with B- and T-ALL, respectively, in animal models [11, 12].

Almost all childhood B-ALL are blocked at the pre-B cell stages of development, where V(D)J rearranged Immunoglobulin heavy (IgH) chains paired with surrogate light chains together with signal transducing immunoglobulin α and β subunits, form the pre-B cell receptor (pre-BCR). The pre-BCR functions as a checkpoint, as only productively rearranged IgH are capable of rescuing cells from clonal extinction mediated through Bach2 induced apoptosis. Rescued pre-B I cells initially undergo a rapid but limited clonal expansion, as large pre-B II cells, before G1 arrest and RAG mediated recombination of light chain genes at the small pre-B II cell stage [12]. Development through these stages involves intricately regulated interaction between transcription factors, metabolic factors, cell surface receptors and the stromal environment, with pre-BCR orchestrated loss of AKT mediated phosphorylation and nuclear translocation of FOXO proteins being one critical effector mechanism (recently reviewed in [13]). Expression of rearranged light and heavy chains on the cell surface along with other signalling molecules, at the immature B-cell stage, results in assembly of B-cell receptors (BCR) that, if productively rearranged and non-auto-reactive, drive further clonal expansion and maturation. Autoreactive BCR produce signals that lead to elimination through PRDM1 dependent premature maturation [14].

To identify 6q TSG contributing to childhood B-ALL, we integrated SNP6.0 array, in-vitro and in-vivo clone tracking assays and expression data, implicating the transcription factors *FOXO3* and *PRDM1*. To investigate the mechanisms by which they suppress leukaemia growth, we combined analysis of gene sets dysregulated by PRDM1, FOXO3 and the related transcription factor FOXO1, with data from a CRISPR-Cas9 knock-out (KO) screen.

## Methods

### Patient material and data

DNA from B-ALL bone marrow samples was obtained from the Blood Cancer UK Childhood Leukaemia Cell Bank or local hospitals. 250 K SNP array files were provided by St Jude Children’s Research Hospital (Table S3). Publicly available whole exome or whole genome sequencing data sets for B-ALL patients (Table S4) were screened for mutations.

### Constructs

The SLIEW lentiviral vector was previously cloned [15] by insertion of a luciferase coding sequence into the *BAMH1* site at position 8256, of pHR-SINcPPT-SIEW (SIN-SIEW) [16]. Consensus coding sequences (CCDS) c-DNA for candidate tumour suppressor genes (IDs - Table S10), synthesised (Life technologies) and cloned into pDONR221, were inserted into the BAMH1 site of a modified SIN-SIEW vector by Gateway^TM^ cloning (Invitrogen-Fisher Scientific). Inserts of resulting SIN-SIEW-cDNA clones were validated by PCR (Table S10) and Sanger sequencing (DBS genomics, Durham, UK).

### Clone tracking experiments

Lentiviral pools were packaged and titrated as previously described [17]. Each pool was used to transduce three cultures of 7.5 × 10^6^ 697 cells, at a multiplicity of infection (MOI) of 0.3. DNA was extracted from 5 × 10^6^ cells at three- or four-day intervals and integrated constructs were amplified and bar-coded with gene specific and universal primers (Table S10). Illumina sequencing was performed by AROS Applied Biotechnology AS (Arhus, Denmark) and reads were aligned to UCSC hg 19 and counted as previously described [18]. Significance of differences in changes of relative counts were calculated by two-way mixed ANOVA in SPSS (v27) (IBM).

For in-vivo assays, 697 cells transduced with lentiviral pools were transplanted intrafemorally into NOD/LtSz-scid IL2Rγ null (NSG) mice. In-vivo imaging, determination of experimental end points and isolation of cells from tissues were performed as previously described [17]. Quantification of constructs in 697 cells from tissues was performed as for in-vitro assays. *p*-values (exact significance) for combined samples, were calculated from the fraction of total read count before/after transplant ratios, for each construct compared with SLIEW, by Mann Whitney U test in SPSS (v27).

To track single constructs in 697 or REH cells, EGFP positive and negative cells were quantified every 3-4 days with an Attune^TM^ NxT Flow Cytometer (Thermo Fisher Scientific, UK). Gating and analysis were performed using FlowJo^TM^ v10.6.2 software (LLC, BD Life Sciences). Statistics were performed as for pooled in-vitro clone tracking experiments.

### Whole genome CRISPR knockout screen

The GeCKO library in the lentiCRISPRv2 backbone, amplified as previously described [19], was a gift from Feng Zhang (Addgene plasmid #52961) [20]. Triplicate cultures of 2.56 × 10^8^ 697 cells were transduced with the packaged library at a MOI of 0.3. Genomic DNA extracted from 3.8 × 10^7^ cells (300-fold library coverage), immediately after puromycin selection (day 0), and weeks 2, 4, 6 and 8, was amplified with custom barcoded primers (Table S11) and sequenced using the Illumina NextSeq 550 platform (Genomics Core Facility, Newcastle University). Reads were aligned to the GeCKO reference sequence, quantified, normalised to non-target reference sequences and analysed statistically using MAGeCK [21] (version 0.5.7). The α-RRA method with 1000 rounds of permutation testing was implemented without removing zero-count sgRNA sequences, sgRNA variance was calculated using all samples, and results were normalised according to copy number status. Target genes were annotated using GRCh37 (hg19).

### Supplementary methods

Details of Cell lines, Western blots, RNA-sequencing, cell cycle assays, analysis of patient deletions and mutations and droplet-digital PCR are provided in supplementary Information.

## Results

### Focal deletions of 6q identify candidate TSG

**Fig. 1:**
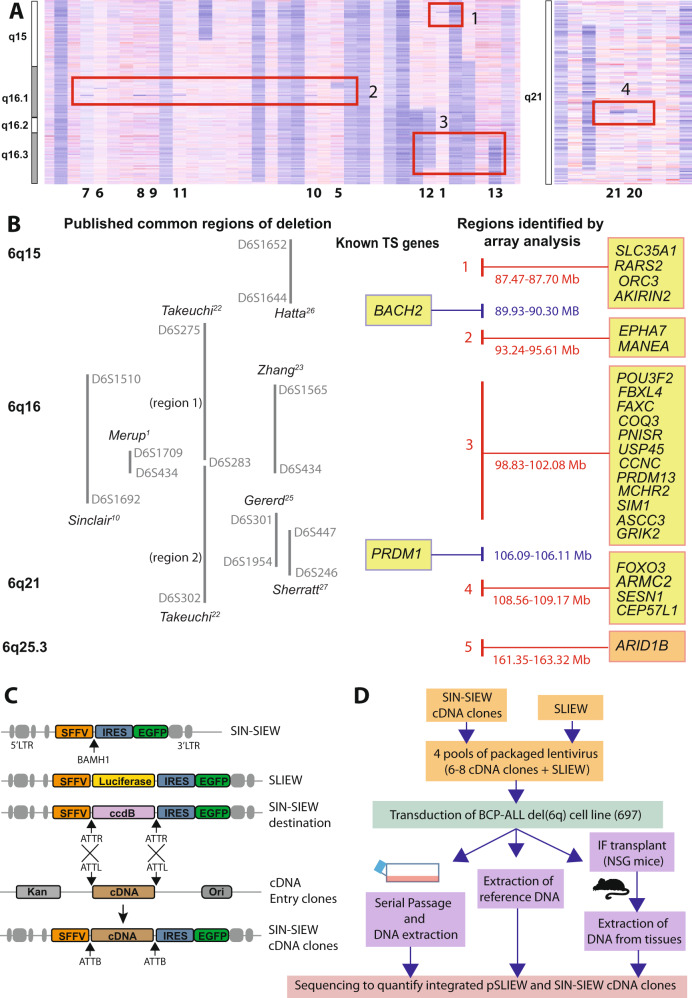
The identification of candidate 6q TS genes. **A** Examples of B-ALL patients with deletions of 6q identified by analysis of 250 K SNP array data. Shades of red and blue in the heat maps indicate SNP probes with a normal and reduced copy number respectively. Red boxes contain examples of focal deletions used to define the boundaries of regions 1–4 shown in B. Corresponding chromosome band positions are as indicated on the left and study specific patient IDs for informative focal deletions (Table S3) are shown beneath the heatmaps. **B** Relative positions of: cytogenetic bands, published common regions of deletion mapped in patients with ALL and recurrent regions of focal deletion based on genomic or SNP array analysis. Green boxes contain symbols for genes within/overlapping the regions of focal deletion and additional genes prioritised for further investigation in functional studies (*BACH2* and *PRDM1*). Red and blue text indicates genomic positions (GRCh38) of deleted regions or candidate tumour suppressor genes, respectively. Region of focal deletion 5, indicated by the orange box containing *ARID1B* only, did not coincide with any previously published common regions of deletion, and was not investigated further in this study. **C** Diagrammatic representation of the lentivirus construct SIN-SIEW showing positions of; the spleen focus forming virus promoter (SFFV), internal ribosomal entry site (IRES) and *EGFP* cDNA. SLIEW, used as a control construct and to facilitate live imaging in-vivo, was constructed by cloning a luciferase cDNA into the indicated BAMH1 site in SIN-SIEW. Using the same BAMH1 site, a ccdB gene flanked by ATTR sites was used to convert SIN-SIEW into a Gateway™ destination vector for recombinational cloning of CCDS (cDNAs). **D** Overview of the methodology involved in clone tracking assays for the identification of functional tumour suppressor genes.

Analysis of in-house and publicly available 250 K SNP and CGH array data identified deletions of 6q in 68 of 440 (15%) patients. Deletions were predominantly large and co-occurred most frequently with *ETV6::RUNX1* (32%) or in T-ALL (22%). Of other well-represented sub-types, the frequency was <13%, and notably low in high hyperdiploid-ALL (4%) (Tables S1 and S2). To define regions of 6q likely to harbour TSG contributing to ALL, we focused specifically on recurrent focal deletions (Table S3 and Fig. 1A). Four of five regions identified were positioned between or within chromosomal bands 6q15 and 6q21 and coincided with one or more previously published CDR derived from ALL or lymphoma patients [1, 10, 22–27]. Although few in number, there was an apparent association between the *ETV6::RUNX1* genetic sub-type and region 3 and 5 deletions, while regions 4 and 2 were predominantly lost in B-other-ALL and high hyperdiploid or T-ALL patients, respectively. No biallelic deletions were identified and non-silent somatically acquired sequence variants within this region of 6q were rare and sporadic, with only 15 reported in 13 genes among 405 B-ALL patients (Table S4). Comparison of published expression data, for genes within or closely adjacent to the focal deletions, in normal early B-cells and three B-ALL cohorts also failed to highlight any individual 6q gene to be repressed in B-ALL (Fig.S1). Therefore all were selected for further functional analysis together with *BACH2* and *PRDM1*, which had been implicated as TSG in lymphoid tumours [6, 12] and are positioned within the 6q15-6q21 deletion hot-spot (Fig. 1B).

### Functional tumour suppressor assays of candidate genes

**Fig. 2:**
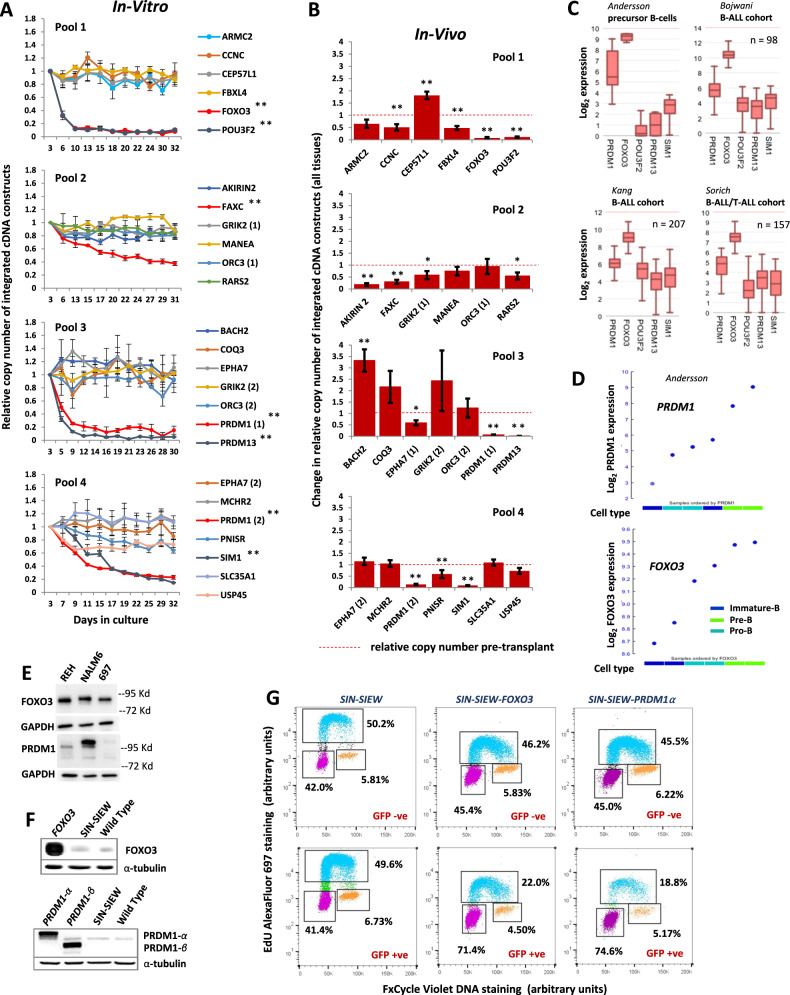
Functional assays and further analysis implicate FOXO3 and PRDM1 as 6q TS genes. **A** Results of pooled in-vitro clone tracking assays in 697 cells. Pools 1 to 4 consisted of equimolar concentrations of SIN-SIEW cDNA constructs expressing the genes indicated together with 20% SLIEW as a control. Numbers 1 and 2 distinguish between different CCDS encoded by a single gene. Construct copy numbers relative to SLIEW at each time point were normalised to the level at day three after transduction. **B** Results of in-vivo clone tracking assays in 697 cells. Histograms, show changes in construct copy numbers relative to SLIEW, in cells purified from tissues after animals had developed leukaemia, compared with pre-transplant levels (indicated by dashed red lines). Data from cells taken from the bone marrow, spleen and liver have been combined and clearly show that expression of genes identified as candidate tumour suppressors in-vitro were also selected against in-vivo. Error bars are standard error of the mean (SEM) and * and ** indicate statistically significant changes in relative copy number of *p* = 0.05–0.01 or <0.01 respectively in both A and B. **C** Relative expression of candidate tumour suppressor genes, identified in 697 cell clone tracking assays, in normal human mixed pro-B, pre-B and immature-B cells (precursor B-cells) and three published ALL cohorts. Highest expression levels were consistently seen for *FOXO3* and *PRDM1*. **D** Comparative expression levels of *PRDM1* and *FOXO3* in normal human pro-B, pre-B and immature-B cells showing a distinctive peak of *PRDM1* expression at the pre-B cell stage. Data sets in **C** and **D** were generated from micro-array experiments archived under GEO accession numbers: Andersson (GSE19599), Bojwani (GSE7440), Kang (GSE11877) and Sorich (GSE10255) and accessed and analysed in the R2 genomics and visualisation platform(https://hgserver1.amc.nl/cgi-bin/r2/main.cgi). **E** Western blot showing evidence for expression of FOXO3 and PRDM1 protein in B-ALL cell lines REH, NALM6 and 697. **F** Western blots showing FOXO3 and PRDM1 protein expression in wild type 697 or in 697 cells sorted for EGFP expression after transduction with the control vector (SIN-SIEW) or SIN-SIEW derived constructs expressing *FOXO3*, *PRDM1α* or *PRDM1β* cDNA. **G** Flow cytometry plots showing proportions of 697 cells in G1 versus S and G2, in EGFP positive and negative populations, 4 days after transduction with SIN-SIEW vector control, SIN-SIEW-FOXO3 and SIN-SIEW-PRDM1α. Replicating cells were detected by a 15 min EdU incorporation, fixation and staining with AlexaFluor-697. DNA content was determined by FxCycle violet stain. Gates show; G1 cells with 2n DNA content and negative EdU staining (purple), S-phase cells with positive EdU staining (cyan) and G2 cells with 4n DNA content and negative EdU staining (orange). In the EGFP + ve fraction of cells transduced with SIN-SIEW-FOXO3 and SIN-SIEW-PRDM1α, but not the empty vector, the percentage of cells in G1 increased while the S-phase populations were depleted.

From among several B-ALL cell lines, we selected 697, which carries a large deletion encompassing 6q15-6q21 and exhibited robust expression of EGFP when transduced with the SIN-SIEW lentivirus vector (Fig. S2). Constructs expressing candidate TSG and EGFP were cloned from SIN-SIEW, while SLIEW [15, 17] served as a control (Fig. 1C). With exceptions of *ASCC3* and *SESN1*, which proved unclonable, SIN-SIEW CCDS constructs were checked for correct sequence insertions then transduced into 697. Indicating efficient expression of upstream candidate TSG, the constructs all expressed EGFP and were used to create assays (Fig. 1D). In-vitro, relative sequence counts remained stable over time for most constructs, while those for *FOXO3*, *POU3F2*, *PRDM13*, *SIM1* and two isoforms (*α* and *β*) of *PRDM1* declined rapidly. Less obvious but significant (*p* < 0.001) negative clonal selection was also seen for *FAXC* (Fig. 2A). Construct levels were also compared between pre-transplant cultures and cells from femurs, livers and spleens of mice transplanted with the same 697 transduced cells (Fig. 2B and S3). Importantly candidate TSG that supressed leukaemia growth in-vitro were also consistently negatively selected in-vivo.

### FOXO3 and PRDM1 are expressed in normal and malignant pre-B cells and induce G1-S arrest

Of genes with strongest TSG activity we detected expression of *FOXO3*, *PRDM1*, *POU3F2* and *PRDM13* by RT-PCR in 697 cells and of all candidates in a second B-ALL cell line, REH (Fig. S4). However we focused further on *PRDM1* and *FOXO3* as the candidate genes most likely to have significant function in pre-B cells, based on their expression in normal and malignant B-cells (Fig. 2C). PRDM1 is recognised as a master regulator of terminal B-cell development (reviewed in [28]) and, although not as highly expressed as in plasma cells, we noted that in early B-cell development, expression peaked between pro-B and immature-B cells at the pre-B cell stage. A modest increase in *FOXO3* expression was also seen at the pre-B cell stage (Fig. 2D). Immunoblots against FOXO3 and PRDM1 confirmed expression in 697 and other B-ALL cell lines (Fig. 2E), as well as markedly increased expression in 697 cells transduced with the expression constructs (Fig. 2F).

We then determined the proportions of 697 cells at different cell cycle stages or showing evidence of apoptosis in EGFP positive and negative populations after transduction with the *FOXO3*, *PRDM1α* or *PRDM1β* expression constructs. Both FOXO3 and PRDM1 induced an increase in the ratio of cells in G1 to S (Fig. 2G) and time course studies indicated both G2-G1 and G1-S phase transitions were delayed by FOXO3 and PRDM1, with G2 progression more markedly affected by PRDM1 (Fig. S5). Over 5 days there was no increase in the proportion of annexin 5 positive cells in EGFP expressing populations (Fig. S6).

### Transcriptomic changes induced by FOXO3 and PRDM1 expression

**Fig. 3:**
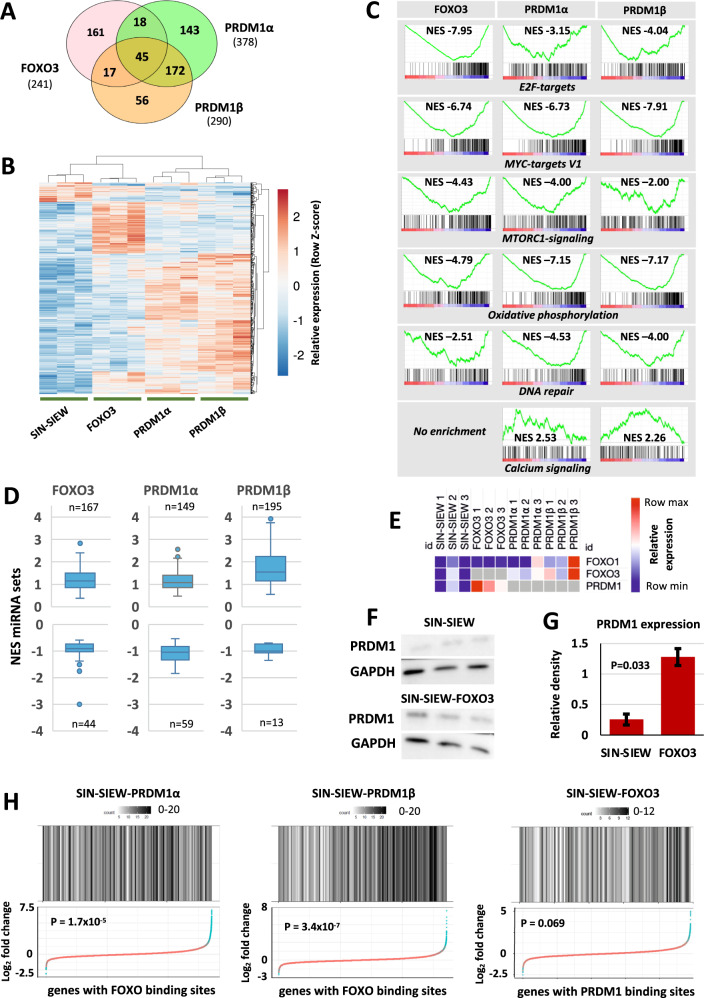
Transcriptomic effects of FOXO3 and PRDM1 expression. **A**, **B** Genes differentially expressed (adjusted *p* < 0.01 and log2 fold change >1) in RNA-sequencing data from 697 cells sorted for EGFP expression after transduction with the control SIN-SIEW vector or constructs expressing *FOXO3*, *PRDM1α* or *PRDM1β* cDNA. **A** Venn diagram, created with VenDiagram in R/Rstudio, illustrating total numbers of genes differentially expressed (adjusted *p* < 0.01, log2 fold change >1) by individual construct (indicated in parenthesis) or by two or all constructs. **B** Heat-map produced in ClustVis showing relative expression, between replicates and expression constructs, of the differentially expressed gene sets. Rows were centred and unit variance scaling was applied to rows. Row and column dendrograms show clustering by correlation distance and average linkage. **C** Examples of GSEA plots of gene sets enriched or depleted by expression of FOXO3 or PRDM1α or β by comparison with SIN-SIEW in 697 cells. Red and blue scaling signifies positive and negative enrichment respectively. Black bars indicate individual gene contributions to the enrichment scores. NES - normalised enrichment score. Further examples of enriched sets and significance of enrichment (nominal *p*-values) are presented in Table S6. **D** Box-and-whisker plots, created in excel, showing the range, median and 1st and 3rd quartiles of NES for gene sets regulated by micro-RNAs in 697 cells transduced with FOXO3, PRDM1α or PRDM1β expression constructs compared with SLEW. Total numbers (n) of positively and negatively enriched sets for each construct are indicated. **E** Heat-map generated in Morpheus showing relative expression of *PRDM1, FOXO3 and FOXO1* in replicate 697 cultures transduced with SIN-SIEW or *FOXO3*/*PRDM1* expression constructs as indicated. Where expression construct and target involve the same gene, data were excluded (grey boxes). **F** Western blots showing consistently increased PRDM1 (BLIMP1) protein levels in 697 cells transduced with the SIN-SIEW-FOXO3 expression construct versus the SIN-SIEW control. **G** Histogram of mean relative expression of PRDM1 protein in 697 cells transduced with SIN-SIEW or SIN-SIEW-FOXO3, as determined by densitometric analysis of Western blots. Error bars are for SEM and the *p*-value for a paired *T*-tests. **H** Dot plots indicate changes in expression of individual genes, with FOXO or PRDM1 binding motifs, induced by *PRDM1α, PRDM1β* or *FOXO3* expression in 697 cells. Blue dots indicate genes with significant fold change in expression (adjusted-*p* < 0.01), heatmaps indicate numbers of genes in expression fold change bins. Expression constructs used are shown in headers and significance of enrichment for dysregulated genes indicated by *p*-values (hypergeometric test). Motif associated gene sets were identified using iRegulon.

RNA-sequencing showed that PRDM1α and PRDM1β induced similar changes in expression profile but also some patterns of dysregulation in common with FOXO3 in 697 cells (Fig. 3A, B). In keeping with roles in promoting pre-BII to immature B-cell maturation and tumour suppression, gene set enrichment analysis (GSEA) demonstrated that all three constructs induced transcriptional changes that were negatively correlated with sets such as the G2M checkpoint, oxidative phosphorylation, glycolysis, fatty acid metabolism, RNA processing and DNA synthesis and repair (Table S6). Downregulation of E2F activity was most strongly associated with FOXO3 expression, while MYC and MTORC1 signalling were suppressed by all three constructs. PRDM1 more potently inhibited oxidative phosphorylation and DNA repair (Fig. 3C), while interestingly PRDM1β compared with PRDM1*α* strongly induced expression of micro-RNA regulated gene sets (*p* < 0.01) (Fig. 3D).

In classical Hodgkin lymphoma, PRDM1 is induced directly by FOXOs [29, 30], so it seemed plausible that the overlapping patterns of gene and pathway dysregulation were in part caused by a similar relationship in pre-B cells. Indeed, in cells expressing *FOXO3*, read counts for *PRDM1* increased significantly (*p* = <0.01), as did protein levels (Figs. 3E–G). Further analysis showed significant enrichment for genes with FOXO binding sites in the *FOXO3* expression set but notably equal or higher enrichment when *PRDM1α* or *β* was expressed (Fig. 3H). Although PRDM1 appeared to modestly induce transcription of both *FOXO3* (p=not significant (NS)) and *FOXO1* (PRDM1*β p* = 0.014, PRDM1*α p* = NS), it seemed unlikely that this alone accounted for the increase in FOXO transcription factor activity. Alternatively, PRDM1 might cooperate with FOXOs as a co-transcription factor or mediate changes in FOXO nuclear localisation or stability. The *FOXO3* expression data set showed a trend towards enrichment for changes in expression of genes with PRDM1 binding sites (*p* = 0.06). From these combined observations, we inferred that PRDM1 and FOXO3 create a positive feed forward loop and that large deletions including 6q21 are likely to have a synergistic damping effect on activity of PRDM1, FOXO3 and possibly other FOXO members.

### FOXO3, PRDM1 and FOXO1 but not BACH2 impair growth of both *TCF3::PBX1* and *ETV6::RUNX1* B-ALL cells

**Fig. 4:**
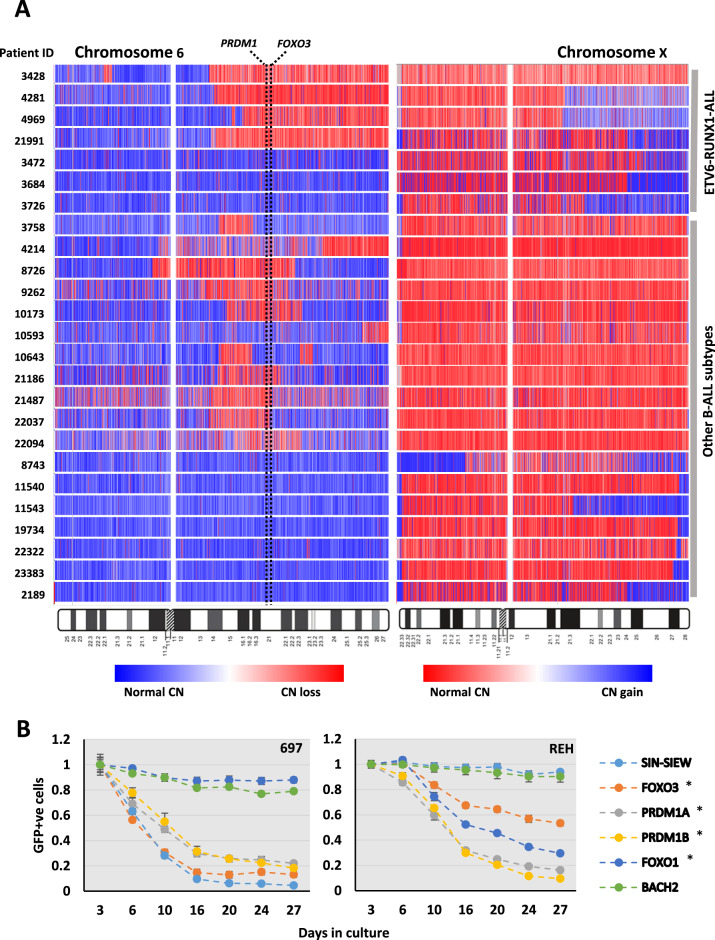
Deletions of 6q and functional clone tracking in *ETV6::RUNX1*-ALL. **A** Copy number (CN) profiles of B-ALL patients with abnormalities of 6q and/or Xq, analysed by SNP6.0 array. In all of four patients bearing *ETV6::RUNX1* fusion and 6q loss, deletions were terminal and co-occurred with terminal amplifications of Xq in three cases. Terminal amplifications of Xq were seen in the absence of 6q deletion in three *ETV6::RUNX1* ALL patients. Terminal and interstitial deletions of 6q or gains of Xq were seen in other B-ALL subtypes but were never co-occurrent in this cohort. Normal CN is indicated by shades of blue or red for chromosomes 6 and X respectively. Dashed vertical lines show the position of *PRDM1* and *FOXO3* and ideograms of chromosomes 6 and X indicate cytogenetic band positions. Patient IDs are indicated to the left of the heatmaps. Heatmaps were generated using log_2_ ratio data in Genotyping Console and rows were scaled individually depending on the clonality of CN abnormalities and sex of patients. **B** Results of single construct clone tracking assays in 697 and REH cells. Percentage of EGFP positive cells, in cultures transduced with single expression constructs, were determined by flow cytometry at each time point and normalised to the level at day three after transduction. Error bars indicate SEM, * indicates divergence from SIN-SIEW in normalised GFP + ve cell counts with time (*p*-value < 0.01) in both cell lines.

In our second cohort of B-ALL patients analysed by SNP6.0 array [31], large deletions of 6q were seen in 4/17 (24%) cases with *ETV6::RUNX1*-positive ALL compared to 15/302 (5%) overall. A review including 164 *ETV6::RUNX1*-ALL cases also reported a high incidence of 6q deletions (20%) [32]. Interestingly three of our four cases, and two published previously [33], co-occurred with amplification of Xq, adding to six reported examples of unbalanced translocations and variable breakpoints, resulting in partial loss of 6q and gain of Xq [der(6)t(X;6)] in *ETV6::RUNX1*-ALL [34–36]. Concurrent loss of 6q and gain of Xq was not seen in any of our patients without *ETV6::RUNX1* fusions and genomic breakpoints from all cases defined a common 6q deleted region between ~100 Mb and the telomere that included *FOXO3* (109 Mb) and *PRDM1* (106 Mb) (Fig. 4A, Tables S7 and S8).

Because expression of genes with FOXO binding motifs were equally or more affected by *PRDM1* compared with *FOXO3* expression (Fig. 3H), we inferred that PRDM1 likely induced more than one FOXO family member. As it contributes to early B cell development [37] and has been implicated as a TSG or as an oncogene in different lymphoma subtypes [38, 39], we investigated the effects of FOXO1 on B-ALL growth. In addition, BACH2 had been reported to function as a TSG in B-ALL [40] but showed no activity in 697 cells, so we tracked levels of SIN-SIEW, *FOXO3*, *FOXO1*, *PRDM1* or *BACH2* as single expression constructs in 697 and the *ETV6::RUNX1*-ALL line, REH, by flow cytometry. As in pooled clone tracking experiments, FOXO3 and PRDM1*α* and PRDM1*β* showed tumour suppressor activity, while BACH2 had no effect on growth of 697. Transduction of REH produced similar results and interestingly FOXO1 also potently supressed growth of both cell lines (Fig. 4B). Therefore, reduced FOXO3 and PRDM1 expression, resulting from 6q deletion, might co-operate both with *TCF3::PBX1* and *ETV6::RUNX1* to promote B-ALL. However, these data also highlight the anomaly that inactivating abnormalities of *FOXO1* are not seen in B-ALL patients.

### Expression of FOXO3 and FOXO1 induces distinct transcriptional programs in 697 cells

**Fig. 5:**
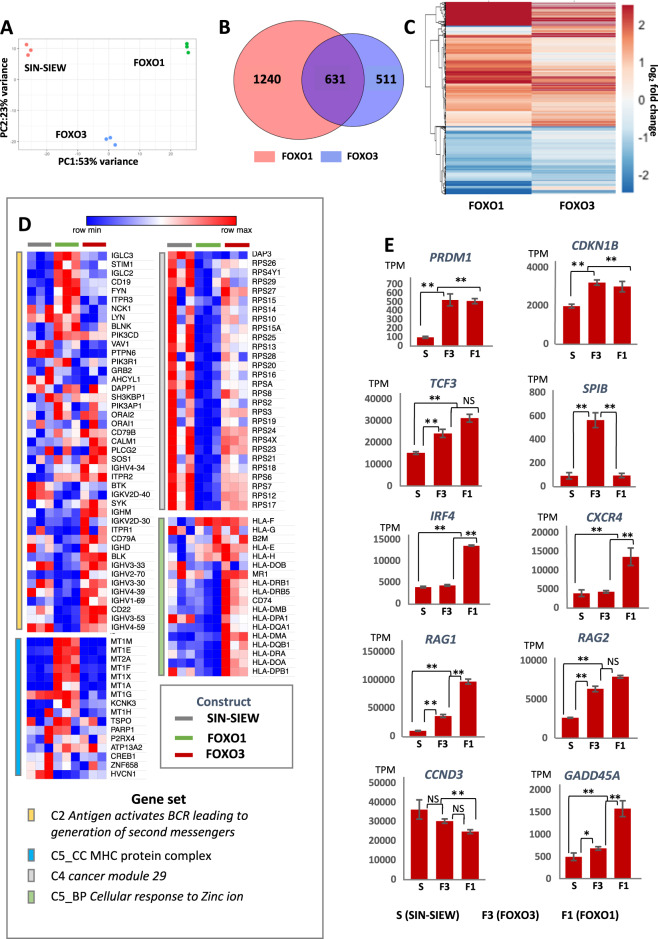
Comparative transcriptomic effects of FOXO3 and FOXO1 expression. **A** Principal component analysis of RNA-sequencing datasets from FACS-sorted replicate 697 cultures transduced with SIN-SIEW, SIN-SIEW-FOXO3 or SIN-SIEW-FOXO1. PC1 – principal component 1; PC2 – principal component 2. **B** Venn diagram illustrating unique and overlapping gene sets differentially expressed (adjusted *p*-value < 0.01, log-2 fold-change >1) by forced expression of *FOXO3* and *FOXO1* in 697 cells. **C** Heat map showing expression relative to control levels of the genes dysregulated by FOXO1 and FOXO3. The scale has been truncated at log_2_-fold-change 2.5 to clearly illustrate the more potent overall effects of FOXO1 expression on transcription levels. Figures A-C were generated in R/RStudio. **D** Heatmaps, generated in Morpheus, showing relative expression, in replicate 697 cultures transduced with SIN-SIEW or FOXO3 / FOXO1 expression constructs, of genes in MSigDB datasets. The examples shown are illustrative of multiple gene sets showing differential expression of; immunoglobulin heavy (*IGHV*) and light (*IGL*) chain genes, small ribosomal subunits (Large ribosomal subunits were also downregulated by FOXO1), HLA class II and metallothionein genes. **E** Histograms showing mean adjusted RNA read counts (TPM) for specific genes involved in early B-cell development for replicate 697 cultures transduced with SIN-SIEW or FOXO expression constructs. Error bars indicate SEM values, significance is indicated by **p < 0.01, *p < 0.05, (*p* > 0.05).

To better understand factors that may account for the contrasting tumour suppressor status of *FOXO3* and *FOXO1* in ALL, we compared RNA-sequencing data from 697 cells transduced with SIN-SIEW-FOXO1, SIN-SIEW-FOXO3 or empty vector. Distinctive roles for FOXO1 and FOXO3 were confirmed by principal component analysis (Fig. 5A) and the size of gene sets commonly and uniquely dysregulated (Fig. 5B). Typically, FOXO1 induced a larger fold change (Fig. 5C). To identify differentially regulated pathways, we performed GSEA with the FOXO1 and FOXO3 data, identifying over 2000 gene sets (*p* < 0.05) (Table S9), including groups of functionally related genes that were also physically clustered (examples in Fig. 5D).

We then analysed individual genes known to contribute to B-cell development. While *PRDM1* was induced strongly by both FOXO1 and FOXO3, other key genes were differentially expressed (Fig. 5E). Notably this applied to the transcription factor, *IRF4*, and chemokine receptor, *CXCR4*, which together create a feedforward loop, critical for niche-driven escape from IL7R signalling, cell cycle exit and establishment of the pre-BII to immature B cell maturation program [41]. While *IRF4* and *CXCR4* were induced solely by FOXO1, of late B-cell transcription factors, *TCF3* expression was elevated by both FOXOs and *SPIB* transcripts were increased 5-fold by FOXO3 but were unaffected by FOXO1. As examples of important genes regulated by the late pre-B cell programme, expression of negative (*CDKN1B*) and positive (*CCND3*) regulators of G1-S phase transition was respectively increased and attenuated by both FOXOs, while *RAG1* and *RAG2*, essential for light chain recombination, were induced robustly by FOXO1 and to a lesser degree by FOXO3, as was *GADD45A*. Taken together, our data confirm that FOXO1 and FOXO3 play partially non-redundant roles in pre-B to immature B-cell maturation. The more stringent block to B-cell development would likely result from loss of FOXO1 which, unlike FOXO3, promoted *IRF4*/*CXCR4* expression and had a more profound effect on induction of the late pre-B cell programme.

### Integrated analysis of data from CRISPR screening and RNA-sequencing highlights functional consequences of PRDM1, FOXO3 and FOXO1 expression

Using data from 697 cells transduced with the whole genome CRISPR knock-out (GeCKO) library, at day 0 and following eight weeks antibiotic selection, we generated two gene sets. The first (*n* = 422) associated with a significant (*p* < 0.05) increase in targeting guide RNAs (gRNA), we classed as having tumour suppressor functions, and the second (*n* = 7843), with significant gRNA depletion (*p* < 0.05), was here considered essential for growth maintenance. Strikingly and in contrast with *FOXO3*, which displayed tumour suppressor activity (*p* = 0.0069), *FOXO1* was ranked second (*p* = 2.41 × 10^−8^) in the list of essential genes, a finding consistent with previous observations that inhibition or knock-down of FOXO1 negatively affects B-ALL growth [42, 43].

**Fig. 6:**
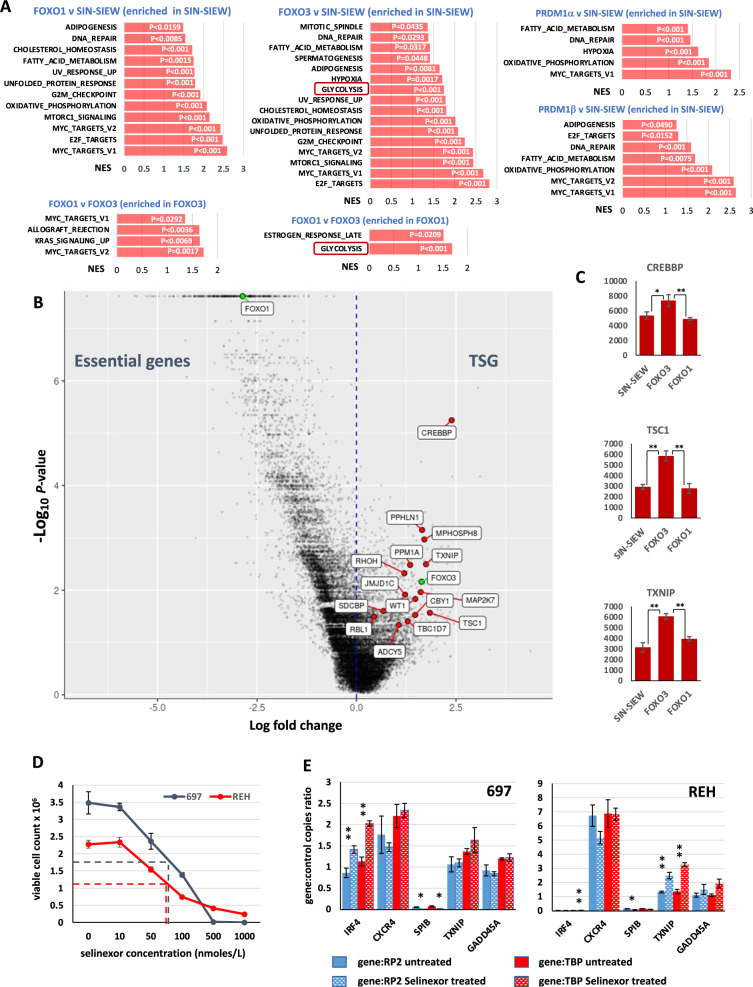
Identification of FOXO3 specific TS functions from a CRISPR-Cas9 screen. Combined analysis of genes with significant changes in guide representation between day 0 and week 8, in a genome wide CRISPR-Cas9 screen of 697, and RNA-sequencing data from 697 cells transduced with SIN-SIEW, SIN-SIEW-FOXO3, SIN-SIEW-FOXO1, SIN-SIEW-PRDM1α or SIN-SIEW-PRDM1β. **A** Genes identified in the CRISPR assay as essential for the maintenance of cell growth (*p* < 0.05), were used to subset the RNA-sequencing data and perform GSEA. Bar plots show normalised enrichment scores (NES) and nominal *p*-values for MSigDB hallmark gene sets enriched (*p* < 0.05) in comparison between replicate cultures transduced with control or expression constructs as indicated. Glycolysis has been highlighted as a key pathway distinguishing between cells expressing FOXO3 versus FOXO1. **B** Volcano plot showing log fold-change and -log_10_ *p*-values for all genes analysed based on guide RNA depletion (negative fold change) or enrichment (positive fold change) in the GeCKO screen. FOXO3 and FOXO1, marked in green, were respectively associated with significant guide enrichment and guide depletion. Genes marked in red were associated with significant guide enrichment and significantly higher expression in cells expressing FOXO3 versus FOXO1. Functionally these genes contribute to cell cycle control; *SDCBP*, *RBL1* and *WT1*, DNA damage response; *PPHLN1* and *MPHOSPH8*, the RAS pathway; *MAP2K7* and *PPM1A*, pre-BCR signalling; *RHOH*, *β*-catenin signalling; *CBY1*, chromatin remodelling; *JMJD1C* and metabolism; *CREBBP*, *TBC1D7*, *TSC1*, and *TXNIP*. As guide enrichment is associated with TSG function, they may contribute to differences in tumour suppressor status of FOXO3 and FOXO1 in B-ALL. **C** Histograms showing mean-adjusted RNA read counts (TPM) for examples of genes associated with guide enrichment and with a known function in the negative regulation of glycolysis. Error bars indicate SEM values and adjusted-*p* values of 0.01–0.05 or <0.01 are indicated by single and double asterisks respectively. **D** Effects on viable cell count of treating 697 and REH cells for 72 h with increasing concentrations of the nuclear export inhibitor Selinexor. As indicated by dashed lines, an IC_50_ in the 50–100 nmolar range was observed for both cell lines. **E** Effects of 48 h treatment with 100 nM Selinexor on gene expression in 697 and REH. Absolute quantification of gene transcripts was determined by droplet digital PCR and gene to control positive droplet copy number ratios are presented for two stably expressed controls, *RP2* and *TBP* as indicated. Treatment significantly induced *IRF4* (697 and REH) and *TXNIP* (REH) but suppressed *SPIB* expression (697 and REH). Expression levels of *IRF4* were notably lower in REH compared with 697. Error bars indicate SEM values. Significant differences in transcript copy between untreated and treated cells, as determined by two tailed *t*-test, are indicated by a single asterisk; *p* = 0.01–0.05, or double asterisk; *p* < 0.01.

We next defined gene sets, enriched or depleted by different expression constructs, among the essential genes (Fig. 6A), revealing that functions and pathways, associated with the large to small B-cell transition, were repressed by FOXO1, FOXO3 and PRDM1. Most highly enriched sets involved MYC and E2F targets, the DNA damage response and metabolism. Notably glycolysis was repressed by FOXO3 (NES 1.74 *p* < 0.001) and PRDM1α (NES 1.05 *p* = NS data not shown) but not by FOXO1 expression. Moreover, glycolysis was one of only two sets enriched in FOXO1 compared with FOXO3 expressing cells. Similar comparison highlighted MYC and KRAS signalling as repressed to a lesser degree by FOXO3, reinforcing evidence that FOXO1 has the more profound influence on large to small pre-B cell transition.

We then focused on genes identified as TSG within the GeCKO screen that were positively regulated by FOXO3 in comparison to FOXO1 (Figs. 6B, C). These included genes involved in cell cycle control, DNA damage response, the RAS pathway, pre-BCR signalling, *β*-catenin signalling and chromatin remodelling. Lastly, and of particular relevance given the emergence of glycolysis as a pathway repressed specifically by FOXO3 expression, several genes contribute to metabolic regulation. *CREBBP*, defined in the CRISPR screen as the second most significant TSG and induced by FOXO3 (*p* = 0.003) and PRDM1 (*p* = 0.009) but unaffected by FOXO1, is an established TSG with both germline and sporadic mutations contributing to B-ALL [44, 45]. Crucially mutations of *CREBBP* in B-ALL promote glycolysis through impaired expression of the glucocorticoid-receptor (GR) responsive genes [46]. B-ALL TS genes PAX5 and IKZF1 activate expression of *NR3C1*, encoding the GR, but also the inhibitor of glycolysis, TXNIP [47], which, in our experiments, was induced by FOXO3 but not by FOXO1. TSC1 also likely plays a role in suppressing glycolysis, as it functions as a molecular co-chaperone, potentially stabilizing both the MTORC1 inhibitor TSC2 and NR3C1 [48].

### Selinexor inhibits growth of B-ALL cells and induces FOXO responsive genes

As increased expression of either FOXO3 or FOXO1 inhibited growth of 697 and REH cells we treated them with Selinexor, a nuclear export inhibitor and FOXO agonist that is licenced for clinical use [49, 50]. IC_50_ were in the low nanomolar range for both cell lines (Fig. 6D) and, consistent with FOXO nuclear retention, expression of key B-cell development genes *IRF4* and *TXNIP* were induced by Selinexor (Fig. 6E).

**Fig. 7:**
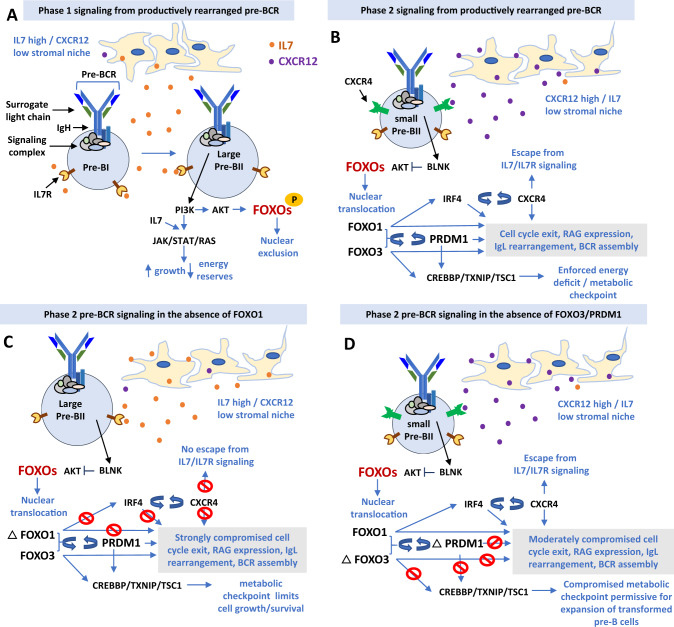
Predicted effects of loss of function of FOXO1 compared with FOXO3/PRDM1 on pre-B cell development. **A** In the first phase of normal pre-B cell development, the newly assembled pre-BCR transduces signals through PI3K which together with IL7R signalling strongly activates the JAK/STAT and RAS pathways, which drive a limited period of clonal expansion of large pre-BII cells. The FOXO transcription factors are inactivated by phosphorylation and nuclear exclusion through PI3K driven activation of AKT. **B** The maturing pre-BCR signalosome activates BLNK which suppresses AKT activity leading to FOXO de-phosphorylation and nuclear translocation. FOXO1 activates the IRF4/CXCR4 feed forward loop resulting in escape from IL7R signalling through cell migration away from the IL7 high/CXCR12 low towards the CXCR12 high/IL7R low stromal niche. Activity of both FOXOs is reinforced by a second feed forward loop involving PRDM1 and these three genes together with CXCR4 contribute to the pre-B to immature B cell maturation program. PRDM1 likely reinforces cell cycle exit permitted by escape from IL7R signalling through displacement of transcription factors such as IRF2 and PAX5 and recruitment of transcriptional repressors such as histone deacetylases HDAC1 and 2 and the histone methyle transferases EHMT2 (G9A) and PRMT5. FOXO3 and PRDM1 also contribute to the maintenance of energy deficit, caused by rapid clonal expansion at the large pre-B cell stage, by inducing genes involved in the suppression of glycolysis. **C** With loss of FOXO1 function (ΔFOXO1), our data predict that IRF4/CXCR4 signalling would be severely compromised, preventing escape from IL7R signalling and initiation of the pre-B to immature B cell maturation program. However, energy deficit, maintained by FOXO3, would prevent unlimited clonal expansion and transformation to leukaemia. **D** With loss of FOXO3 and PRDM1 function (ΔFOXO3/ΔPRDM1) through deletion of chromosome 6, although the IRF4/CXCR4 axis would remain largely intact, there would be some effect on the normal pre-B cell transcriptional program. Crucially, suppression of glycolysis and maintenance of energy deficit would be compromised, contributing to an environment permissive for continued cell growth and leukemic transformation.

Based on our whole transcriptome sequencing, functional screening with CRISPR-cas9, response to Selinexor, the known Pre-BII to immature B cell metabolic checkpoint [47] and PRDM1 functions in transcriptional repression (reviewed in [51]), we propose a model to explain the role of 6q deletions in B-ALL (Fig. 7).

## Discussion

FOXO activity is controlled by transcriptional regulation, micro-RNA mediated degradation and post-translational modifications at several sites mediated by multiple signalling molecules. Modifications can alter potential to affect target gene expression or promote nuclear export and degradation (reviewed in [52, 53]). Although consensus DNA sequences bound by FOXOs are the same, differences in transactivation domains mean that potential to be regulated through modification and to interact with partner proteins differ between family members [54]. Our data highlight clear differences in the transcriptional programmes mediated by FOXO1 and FOXO3, suggesting that appropriate regulation of cellular localisation, stability, abundance and modifications of the two proteins is required for normal early B-cell development. At the functional level, it was striking that elevated expression of both FOXOs inhibited B-ALL growth but, while even on a background of pre-existing haploinsufficiency loss of FOXO3 function was advantageous, knock-out of FOXO1 was heavily selected against. A similar bimodal response to activation/inhibition of FOXO1 in the context of B-ALL has been reported previously and counterintuitively, while our RNA-sequencing data provided ample evidence that FOXO1 overexpression reduced MYC, MTORC1 and CCND3 activity, these functions were also suppressed by FOXO1 inhibition [43]. FOXO1 inhibition has also been shown to suppress the DNA damage response in-vitro and disease burden in B-ALL patient derived xenografts [42]. Pharmacological modulation of FOXO activity has been achieved through a variety of routes [53]. As both FOXO3 and FOXO1 inhibited leukaemia growth in our assays, we treated 697 and REH cells with Selinexor to increase pan-FOXO activity [49, 50, 55], achieving IC_50_ in the 50–100 nanomolar range. As T-ALL cells showed IC_50_ of 34–203 nanomolar and in pre-clinical studies responded to Selinexor, at a dose showing minimal adverse effects and absence of toxicity to normal blood cells [56], we demonstrate a potential therapeutic window for the treatment of B-ALL. Other agents repress PI3K and/or mTOR, leading to reduced AKT activity and consequent FOXO inactivation through phosphorylation. Our data also indicate that in B-ALL, deletions of 6q may flag a metabolic vulnerability, which could be exploited by specific activation of FOXO3 or treatment with inhibitors of glycolysis such as the TXNIP agonist 3-O-methyleglucose [57]. Lastly, *PRDM1* expression was induced by both FOXOs and strongly selected against in clone tracking assays. In mouse models, fasting induced leptin receptor activity was shown to promote B- and T-ALL differentiation and reduce leukaemia burden in a manner dependent on induction of PRDM1 [58]. Deletions of 6q involving *FOXO3* and/or *PRDM1* may therefore promote leukaemia survival partly by reducing sensitivity to leptin receptor signalling. In the absence of PRDM1 agonists, dietary restriction or FOXO activation would feasibly elevate *PRDM1* expression from the remaining allele to levels where leukaemia cells differentiate.

In this study clone tracking assays were limited to specific ALL subtypes and, although other functional candidates were identified, we further focused on genes showing highest expression in normal pre-B or B-ALL cells. Although currently without known function in B-cells, It is important to acknowledge that loss of *POU3F2*, *PRDM13*, *SIM1* and *FAXC* may also contribute to the leukemogenic potential of large 6q deletions. *CCNC*, and *BACH2* showed no TS potential in this study but have been implicated in other sub-types of ALL [11, 12] and two genes, *SESN1* and *ASCC3*, within focal deleted regions remained untested for technical reasons. *SESN1* is co-regulated with *FOXO3* [59] and is a target of the EZH2^Y641X^ gain of function mutation and of focal deletions in follicular lymphoma [60]. Loss of SESN1 function disrupted p53 mediated suppression of mTORC1 mRNA translation suggesting potential functional cooperation with FOXO3 in controlling energy balance and suppression of leukemia.

In conclusion, we identified two candidate TSG through functional analysis on a *TCF3::PBX1* background. Our preliminary patient screen suggests that other genes in the region contributing to ALL will be revealed by larger scale genomic studies and functional analysis of other sub-types. We found that REH cells harbouring the *ETV6::RUNX* fusion but no del(6q) were also vulnerable to over-expression of *FOXO3, PRDM1* and *FOXO1* suggesting therapeutic relevance of pathways dysregulated by these genes for a range of ALL patients. Comparison of FOXO3 and FOXO1 induced transcriptomic changes underline the importance of the metabolic checkpoint in B-ALL maintenance and potential value of screening patient-derived blasts for response to agents such as FOXO agonists or inhibitors of glycolysis. In the future, it may also be possible to leverage data from whole genome-CRISPR or other functional screens, performed on patient derived material, to identify ALL subtypes potentially responsive to manipulation of the FOXO-PRDM1 axis.

## Supplementary information

supplementary materials

Large supplementary tables

## Data Availability

RNA-sequencing data sets generated and analysed during the current study are available through GEO (accession number GSE193349). For original CRISPR screening data please contact Ruth Cranston (Ruth.Cranston@newcastle.ac.uk).
