# Supplementary material for: Disruption to the FOXO-PRDM1 axis resulting from deletions of chromosome 6 in acute lymphoblastic leukaemia
Source: Leukemia. 2023 Jan 20;37(3):636–49. doi: 10.1038/s41375-023-01816-0 (PMC9991907; doi:10.1038/s41375-023-01816-0)
Supplement: Supplementary file 1 — supplementary materials [file 41375_2023_1816_MOESM1_ESM.pdf]

Supplementary methods.

Cell lines. Cell lines were obtained from the DSMZ-German collection of Microorganisms and Cell Cultures and were maintained in RPMI medium with 10 or 20% foetal calf serum at cell densities recommended by DSMZ. Transduction with SIN-SIEW lentivirus was used to assess lymphoid cell lines with del(6q); 697, RPMI 8402, MOLT 4, PEER, and MN-60 and without 6q abnormalities NALM-6, RCH-ACV, REH, SEM and BV-173. Based on superior transduction efficiency and level of GFP expression, 697 and REH were selected for clone tracking assays. The authenticity of 697 and REH was checked by DNA fingerprint on an annual basis

Analysis of patient deletions and mutations. To identify regions of 6q deletion, DNA from 135 B-ALL bone marrow samples, obtained from the Blood Cancer UK Childhood Leukaemia Cell Bank or local hospitals (Newcastle cohort 1), was hybridized to genomic arrays and analyzed as described.[1,2] Additional 250K SNP array paired diagnostic and remission ALL-patient (n= 305) and normal control (n = 62) files were provided by St Jude Children's Research Hospital[3,4] and analysed with the Partek Genomics Suite v6.5 (Partek, USA). Breakpoints of deletions were defined by visual inspection of chromosome 6 copy number (CN) heat maps and scatter plots and were designated as somatically acquired and leukemia related if not present in the remission sample or normal controls. Core regions of recurrent focal deletion were identified by overlap of small deletions and boundaries of regions 1-5 defined by the 5' and 3' ends of any genes potentially affected by the core deletions, through loss of coding sequences, regulatory elements or insulator sequences (Table S3). Large deletions of 6q or amplifications of Xq (Table S8) were identified by analysing SNP6.0 array data from a second cohort of diagnostic B-ALL samples (Newcastle cohort 2) as previously described.[5] Unpublished and publicly available whole exome or whole genome sequencing data sets for B-ALL patients[6-12] (Table S4) were screened for mutations affecting protein coding genes between 87.4 and 109.2 Mb of chromosome 6.

RNA sequencing. Cells were sorted for EGFP expression 4 days after transduction (Flow Cytometry Core Facility, Newcastle University), using a FACSAria III flow cytometer (BD Biosciences). RNA sequencing was performed either; (experiment 1) using the Illumina HiSeq 2000/2500 platform (~90 million 100bp paired-end reads per sample) (AROS, Applied Biotechnologies, Arhus, Denmark) or (experiment 2) the Illumina NextSeq 500 platform (~30 million 70bp paired-end reads per sample) (Genomics Core Facility, Newcastle University). Reads were aligned to hg19 (UCSC) in experiment 1 with STAR[13] then quantified and counted by mapping to the GENCODE (v17) reference sequence using HT-Seq[14]. In experiment 2, reads were aligned, mapped (GENCODE v34) and counted using SALMON[15]. Gene set enrichment analysis (GSEA)[16,17] was performed in experiment 1 with GSEA (v3) using pre-ranked counts and the Molecular Signatures Database (MSigDB) (v5) gene sets and in experiment 2 with GSEA (v4.1.0) and MSigDB (v7.4) gene sets without pre-ranking.
All histograms were created in Excel (Microsoft corporation) Additional software used to generate figures included; R/RStudio (http://www.rstudio.com/), ClustVis[18], Morpheus (https://software.broadinstitute.org/morpheus), and iRegulon[19] as indicated.
RNAseq data are available through GEO (accession number GSE193349).

Western blots, Cell cycle and apoptosis assays. Antibodies used for Western blots are presented in Table S5. Images were captured with a Synegene G:Box Chemi XL 1.4 (Cambridge, UK). For detection of apoptosis, cells were stained using a GFP-certified Apoptosis/Necrosis Detection Kit (Enzo life sciences, inc.) and analysed using a BD FACSCanto II cell analyser and BD FACSDiva software (BD Biosciences). For cell cycle analysis, I ml 697 cells/well were plated at 1x10^6/ml and treated the following day with 10um EdU for between 1 and 10 hours or with 30um EdU for 15 minutes. Treated cells were fixed in paraformaldehyde, permeabilised and stained for EdU incorporation with Alexa Flour 647 azide using a Click-iT EdU Alexa Fluor 647 flow cytometry assay kit (ThermoFisher Scientific

#C10424) following the manufacturers instructions. Washed cells were further stained for DNA content with 1ul/ml FxCycle Violet (ThermoFisher Scientific #F10347) before Flow analysis using a Fortessa systems flow cytometer and laser/ bandpass filter combinations; 488/530 (EGFP), 635/670 (Alexa Fluor 647) and 405/450 (FxCycle Violet). 50,000 cells were analysed in total per sample and gating and further analysis was carried out using FlowJo™ v10.6.2 software (LLC, BD Life Sciences).

Selinexor treatment. Selinexor (KPT-330)(Selleckchem.com, TX USA) was dissolved in DMSO to 10mM and diluted in RPMI medium with 10% foetal calf serum to final concentrations of 0.01, 0.05, 0.1 1.0 μM. 697 and REH cells were resuspended at 0.5 or 1.0 x10^6 cells/ml respectively in media containing Selinexor dilutions or vehicle only control media (DMSO 1:10,000) and plated as 0.5ml cultures in 24 well plates. Three replicate wells with vehicle control media and each Selinexor dilution were cultured for 72 hours before staining with trypan blue and manually counting viable cells.
To assess effects of Selinexor on expression of B-cell development genes, 5ml cultures of 697 and REH resuspended in media containing vehicle control or 0.1 μM Selinexor were cultured for 48 hours. 3x10^6 cells taken from each of four replicate cultures of control and Selinexor treated cells were used for RNA preparations.

RT-PCR and quantitative droplet digital PCR. RNA was prepared from 697 and REH cells using a RNeasy kit (Qiagen, UK). cDNA was prepared from 200ng RNA from each culture with a QuantiTect Reverse Transcriptase kit (Qiagen). Candidate 6q TS genes were analysed by PCR using 1μl of 1:20 dilutions of 697 or REH cDNA and NT control, 1ul primer pair (50nM), and 10μl QX200ddPCR EvaGreen supermix (Bio-Rad,UK) in 20μl reactions. Reactions were cycled at; 95°C 5min, 40x (96°C 30sec, 58°C 1min), 4°C 5min, 90°C 5min, 4°C infinity. Primer pairs and expected PCR product sizes are presented in supplementary table 12. Genomic human DNA (50ng/reaction) was used as a positive control and non-template (NT) negative controls were prepared from the RNA stocks, as for cDNA but without addition of reverse transcriptase.
Expression of B-cell development genes in control and Selinexor treated cells was assessed by droplet-digital-PCR (ddPCR). Primer pairs targeting *IRF4*, *CXCR4*, *SPIB*, *TXNIP* and *GADD45A* and two controls *RP2* and *TBP* were initially tested by RT-PCR in 20ul reactions, as for the 6q candidate TS genes. A single primer pair for each gene was selected (supplementary table 13) and used in the same 20μl reactions in 96 well plate format with 1ul undiluted cDNA or NT control from 4 experimental replicates of untreated or Selinexor treated 697 or REH cells. Droplet generation, PCR and droplet reading were performed respectively with; a Bio-Rad AutoDG instrument, a C100 Touch Thermal Cycler, using the cycles described for RT-PCR, and a QX200 Droplet Digital PCR System (Bio-Rad). Absolute quantification of positive droplets was performed with QuantaSoft Analysis Pro software (Bio-Rad), using manual thresholds, and data was exported to Excel. Zero or negligible positive droplets were recorded for NT controls. Mean and SEM of ratios of numbers of positive droplets for target genes and each control, *RP2* and *TBP*, were calculated for the four experimental replicates in treated and untreated groups. *p*-values for differences in the mean ratios between untreated and selinexor treated cultures for each target gene and cell line were calculated by 2-tailed student t-test. Calculations were performed and charts made in Excel.

References.

1. Russell LJ, Capasso M, Vater I, et al. Deregulated expression of cytokine receptor gene, CRLF2, is involved in lymphoid transformation in B-cell precursor acute lymphoblastic leukemia. *Blood*. 2009;114(13):2688-2698.

2. Rand V, Parker H, Russell LJ, et al. Genomic characterization implicates iAMP21 as a likely primary genetic event in childhood B-cell precursor acute lymphoblastic leukemia. *Blood*.
3. Mullighan CG, Goorha S, Radtke I, et al. Genome-wide analysis of genetic alterations in acute lymphoblastic leukaemia. *Nature*. 2007;446(7137):758-764.
4. Mullighan CG, Miller CB, Radtke I, et al. BCR-ABL1 lymphoblastic leukaemia is characterized by the deletion of Ikaros. *Nature*. 2008;453(7191):110-114.
5. Sinclair PB, Ryan S, Bashton M, et al. SH2B3 inactivation through CN-LOH 12q is uniquely associated with B-cell precursor ALL with iAMP21 or other chromosome 21 gain. *Leukemia*. 2019;33(8):1881-1894.
6. Holmfeldt L, Wei L, Diaz-Flores E, et al. The genomic landscape of hypodiploid acute lymphoblastic leukemia. *Nat Genet*. 2013;45(3):242-252.
7. Roberts KG, Li Y, Payne-Turner D, et al. Targetable kinase-activating lesions in Ph-like acute lymphoblastic leukemia. *N Engl J Med*. 2014;371(11):1005-1015.
8. Papaemmanuil E, Rapado I, Li Y, et al. RAG-mediated recombination is the predominant driver of oncogenic rearrangement in ETV6-RUNX1 acute lymphoblastic leukemia. *Nat Genet*. 2014;46(2):116-125.
9. Paulsson K, Lilljebjorn H, Biloglav A, et al. The genomic landscape of high hyperdiploid childhood acute lymphoblastic leukemia. *Nat Genet*. 2015;47(6):672-676.
10. Andersson AK, Ma J, Wang J, et al. The landscape of somatic mutations in infant MLL-rearranged acute lymphoblastic leukemias. *Nat Genet*. 2015;47(4):330-337.
11. Ryan SL, Matheson E, Grossmann V, et al. The role of the RAS pathway in iAMP21-ALL. *Leukemia*. 2016;30(9):1824-1831.
12. Russell LJ, Jones L, Enshaei A, et al. Characterisation of the genomic landscape of CRLF2-rearranged acute lymphoblastic leukemia. *Genes Chromosomes Cancer*. 2017;56(5):363-372.
13. Dobin A, Davis CA, Schlesinger F, et al. STAR: ultrafast universal RNA-seq aligner. *Bioinformatics*. 2013;29(1):15-21.
14. Anders S, Pyl PT, Huber W. HTSeq--a Python framework to work with high-throughput sequencing data. *Bioinformatics*. 2015;31(2):166-169.
15. Patro R, Duggal G, Love MI, Irizarry RA, Kingsford C. Salmon provides fast and bias-aware quantification of transcript expression. *Nat Methods*. 2017;14(4):417-419.
16. Subramanian A, Tamayo P, Mootha VK, et al. Gene set enrichment analysis: a knowledge-based approach for interpreting genome-wide expression profiles. *Proc Natl Acad Sci U S A*. 2005;102(43):15545-15550.
17. Mootha VK, Lindgren CM, Eriksson KF, et al. PGC-1alpha-responsive genes involved in oxidative phosphorylation are coordinately downregulated in human diabetes. *Nat Genet*. 2003;34(3):267-273.
18. Metsalu T, Vilo J. ClustVis: a web tool for visualizing clustering of multivariate data using Principal Component Analysis and heatmap. *Nucleic Acids Res*. 2015;43(W1):W566-570.
19. Janky R, Verfaillie A, Imrichova H, et al. iRegulon: from a gene list to a gene regulatory network using large motif and track collections. *PLoS Comput Biol*. 2014;10(7):e1003731.

Supplementary Tables.

Table S1. Frequency by genetic sub-type of 6q deletions of the St Jude's cohort analysed by 250K SNP array.

Table S1. Frequency by genetic sub-type of 6q deletions of th[illegible]			
**Subtype**	**#patients**	**# with del(6q)**	**% with del(6q)**
BCR::ABL1	43	5	11.6
TCF3::PBX1	17	2	11.7
Hyperdiploid 47-50	24	2	8.3
Hyperdiploid 50	40	1	2.5
Hypodiploid	10	1	10
KMT2A	23	2	8.7
other	26	1	3.8
Pseudodiploid	24	4	16
T-ALL	50	10	20

Table S2 Frequency by genetic sub-type of 6q deletions in Newcastle cohort 1 analysed by aCGH.

Subtype	#patients	# with del (6q)	% with del(6q)
B-other-ALL	53	7	12.9
ETV6::RUNX1	36	15	41.6
iAMP21-ALL	24	2	8.3
T-ALL	12	4	33.3
Hyperdiploid	5	0	0
Hypodiploid	4	0	0
DS-ALL	1	0	0
Overall	135	28	20.7

Table S3 is in the supplementary large tables excel file.

Table S4. Mutations* identified in B-ALL patients in coding sequence or UTRs of genes in the 6q15-21 region.

Study	# of patients	sub-type	patient ID	gene	genomic position	mutation
Holmfeldt[6]	40	hypodiploid	SJHYPO054-D	EPHA7	6:93,240,020-93,419,687	Del (e3-DS)
		hypodiploid	SJHYPO088-D	EPHA7	6:93,240,020-93,419,687	Del (3' UTR-DS)
		hypodiploid	SJHYPO006-D	PRDM1	6:105,993,463-106,109,939	SNV p.E7_UTR_3
		hypodiploid	SJHYPO001-D	SLC22A16	6:110,424,587-110,476,674	indel p.S275fs
		hypodiploid	SJHYPO124-D	HDAC2	6:113,933,028-114,011,308	SNV p.S118P
Roberts[7]	154	BCR-ABL-like	SJBALL021486	FHL5	6:96,562,548-96,618,626	SNV E1_UTR_5
		BCR-ABL-like	SJHYPO110	PRDM1	6:105,993,463-106,109,939	SNV E1_UTR_5
		BCR-ABL-like	SJBALL020877	ATG5	6:106,045,423-106,325,791	SNV p.L77R
		BCR-ABL-like	SJBALL020936	DDO	6:110,388,321-110,415,575	SNV p.P291S
		BCR-ABL-like	SJBALL021305	HS3ST5	6:114,055,586-114,343,023	SNV p.A7V
Papaemmanuil[8]	56	ETV6-RUNX1	PD4035a	GABRR1	6:89,177,504-89,231,288	SNV p.R63Q
		ETV6-RUNX2	PD4020a	MDN1	6:89,642,498-89,828,021	SNV p.P2673A
		ETV6-RUNX3	PD4036a	FBXL4	6:98,868,535-98,948,006	SNV p.L433V
		ETV6-RUNX4	PD4009a	GRIK2	6:100,962,701-102,081,622	SNV p.F167L
		ETV6-RUNX5	PD4020a	ARMC2	6:108,848,410-108,989,090	SNV p.R107T
Paulsson[9]	51	High hyperdiploid	No mutations reported			
Andersson[10]	85	MLL rearranged	No mutations reported			
Ryan[11]	8	iAMP21	No mutations reported			
Russell[12]	11	CRLF2 rearranged	No mutations reported			

*Non -silent coding region, splice site or UTR mutations in protein coding genes only are presented.

References supplied in supplementary reference list.

Table S5. Antibodies used for Western blots.

Antibody	primary/secondary	manufacturer / ID	Dilution
rabbit monoclonal anti-BLIMP1	primary	Cell signalling technology / 9115	1:1000
rabbit monoclonal anti-FOXO3	primary	Cell signalling technology / 2497	1:2000
HPR conjugated anti-rabbit IgG	secondary	Cell signalling technology / 7074	1:2000
mouse monoclonal HPR conjugated anti-GAPDH	primary	ABCAM / ab9482	1:15000

Table S6 is in the supplementary large tables excel file.

Table S7. Cases of *ETV6::RUNX1* ALL identified in this study and previously published with concurrent del(6q) / amp(Xp).

Cases of ETV6-RUNX1 ALL with concurrent 6q deletion / Xq amplification presented in this study				
**RegID**	**technique**	**region of 6q deletion (CRCh38)**	**region of Xq amplification (CRCh38)**	**Sex**
21991	SNP6	82-171 Mb	123-155 Mb	M
4281*	SNP6/aCGH	82-171 Mb	90-155 Mb	M
4969*	SNP6/aCGH	90-92, 96-171 Mb	88-155 Mb	M
4013*	aCGH	100-171 Mb	123-155 Mb	F
4250*	aCGH	91-171 Mb	129-155 Mb	M
**Published cases of ETV6-RUNX1 ALL with der(6)t(6;X)**				
**ID/ref**	**technique**	**region of 6q deletion (CRCh38)**	**region of Xq amplification (CRCh38)**	**Sex**
(1)[34]	aCGH/WCP	96-171 Mb	96-155 Mb	M
(2)[34]	aCGH/WCP	83-171 Mb	83-155 Mb	F
(3)[34]	aCGH/WCP	95-171 Mb	122-155 Mb	M
(2)[36]	WCP	q13-q14-qtel	q21-q22-qtel	F
(1)[35]	aCGH/WCP	82-171 Mb	89-155 Mb	M
(8)[35]	aCGH/WCP	82-171 Mb	87-155 Mb	M

aCGH (array comparative genomic hybridisation)
WCP (whole chromosome paint)
*aCGH data for these cases was previously published[33]
References supplied in the main text

Table S8. Genomic regions of copy number loss of 6q and gain of Xq in B-ALL patients (Newcastle Cohort 2).

Patient ID	6q deletion	Genomic position (hg19)	Xq amplification	Genomic position (hg19)	M/F	Sub-type
3428*	del(6q)	78.7Mb-tel			M	ETV6-RUNX1
4281*	del(6q)	81.6Mb-tel	amp(Xq)	89.8Mb-tel	M	ETV6-RUNX1
4969*	del(6q)	90.3-92.2Mb/95.8Mb-tel	amp(Xq)	87.8Mb-tel	M	ETV6-RUNX1
21991	del(6q) sub-clonal	82.0Mb-tel	amp(Xq) sub clonal	123.3Mb-tel	M	ETV6-RUNX1
3472			amp(Xq) sub-clonal	129.5Mb-tel	F	ETV6-RUNX1
3684			amp(Xq) sub-clonal	123.1Mb-tel	F	ETV6-RUNX1
3726			amp(Xq) sub-clonal	99.9Mb-tel	M	ETV6-RUNX1
3758	del(6q)	83.8-101.1Mb			M	B-other
4214	del(6q) sub-clonal	136.9Mb-tel			M	t(8;22) IGH rearranged
8726	del(6q)	49.9-122.7Mb			F	PAX5-ETV6
9262	del(6q) sub-clonal	74.5-118.2Mb			F	B-other ERG deleted, CDKN1A deleted
10173	del(6q)	87.9-126.4Mb			M	B-other
10593	del(6q)	156.9Mb-tel			M	PAX5-ARHGAP22 / complex karyotype
10643	del(6q), del(6q)	83.8-100.7Mb/125.2-131.5Mb			F	hyperdiploid
21186	del(6q) sub-clonal	83.4-116.0Mb			M	IGH-ID4
21487	del(6q) sub-clonal	79.8-110.8Mb			F	IKZF1 N159Y
22037	del(6q) sub-clonal	78.9-109.9Mb			F	IGH-DUX4
22094	del(6q) sub-clonal	90.9-125.0Mb			F	EP300-ZNF384
8743*			amp(Xp),amp(Xq)	tel-37.1Mb / 123.1Mb-tel	F	iAMP21
11540			amp(Xq)	150.4Mb-tel	F	IGH-CEBPA
11543			amp(Xq)	93.7Mb-tel	M	IGH-CRLF2
19734			amp(Xq)	149.7Mb-tel	M	IGH rearranged
22322			amp(Xq)	148.6Mb-151.3Mb	F	iAMP21
23383			amp(Xq)	147.0Mb-tel	F	iAMP21
2189*			amp(Xq) sub-clonal	123.1Mb-tel	M	B-other

*Analysed by aCGH (Newcastle cohort 1)

Table S9 is in the supplementary large tables excel file.

Table S10. Gene names, nucleotide and CCDS accession numbers and primers used for sequencing in clone tracking assays.

Gene ID	CCDS number	Nucleotide ID	CCDS ID	Reverse primer for preparation of sequencing libraries.
SLC35A1	1	NM_006416	CCDS5010	TACGGTAGCAGAGACTTGGTCTCCATCAGGGTCATCACTGC
RARS2	1	NM_020320	CCDS5011	TACGGTAGCAGAGACTTGGTCTGGAAAGCTGGCAAGCAATAG
ORC3	1	NM_181837	CCDS5012	TACGGTAGCAGAGACTTGGTCTTGGCTTAAAAACAAAGCAACC
ORC3	2	NM_012381	CCDS43486	TACGGTAGCAGAGACTTGGTCTTGGCTTAAAAACAAAGCAACC
AKIRIN2	1	NM_018064	CCDS5013	TACGGTAGCAGAGACTTGGTCTGGGTCGAAATCCAGAGTCCT
BACH2	1	NM_021813	CCDS5026	TACGGTAGCAGAGACTTGGTCTGTGCAGTGGACTGTGGACTC
EPHA7	1	NM_004440	CCDS5031	TACGGTAGCAGAGACTTGGTCTCTCCCCTGTGTGTGCAAAG
EPHA7	2	NM_001288630	CCDS75494	TACGGTAGCAGAGACTTGGTCTCTCCCCTGTGTGTGCAAAG
MANEA	1	NM_024641	CCDS5032	TACGGTAGCAGAGACTTGGTCTAAAGTGCCAAAATGATGCAA
POU3F2	1	NM_005604	CCDS5040	TACGGTAGCAGAGACTTGGTCT AGGTGAGCAGGCTGTAGTGG
FBXL4	1	NM_012160	CCDS5041	TACGGTAGCAGAGACTTGGTCTTTCTCCTCTTGTAGCTGTCCTG
FAXC	1	NM_032511	CCDS34500	TACGGTAGCAGAGACTTGGTCTCGAAGAAGGAGATGCTCTGG
COQ3	1	NM_017421	CCDS5042	TACGGTAGCAGAGACTTGGTCTGACGCGCAGCTTTTGTATTA
PNISR	1	NM_032870	CCDS5043	TACGGTAGCAGAGACTTGGTCTGATCCTGTTGGTGCTGGAAT
USP45	1	NM_001080481	CCDS34501	TACGGTAGCAGAGACTTGGTCTTCATGAGGTACAGTAGGCCTTT
CCNC	1	NM_005190	CCDS34502	TACGGTAGCAGAGACTTGGTCTTAAATCCTTTTGGCGCTCCT
PRDM13	1	NM_021620	CCDS43487	TACGGTAGCAGAGACTTGGTCTAGGTACTTGCCCAGCTTGAA
MCHR2	1	NM_032503	CCDS5044	TACGGTAGCAGAGACTTGGTCTCACTGGCAGTTTGATAAGCA
SIM1	1	NM_005068	CCDS5045	TACGGTAGCAGAGACTTGGTCTGGGCAAAGGCAGTAATTTAGC
GRIK2	1	NM_021956	CCDS5048	TACGGTAGCAGAGACTTGGTCTCCAATCCACAGTAAACAGAGCA
GRIK2	2	NM_175768	CCDS5049	TACGGTAGCAGAGACTTGGTCTCCAATCCACAGTAAACAGAGCA
PRDM1	1	NM_001198	CCDS5054	TACGGTAGCAGAGACTTGGTCTCCCTGAAACCTCACAGTGCT
PRDM1	2	NM_182907	CCDS34505	TACGGTAGCAGAGACTTGGTCTATAGCGCATCCAGTTGCTTT
FOXO3	1	NM_201559	CCDS5068	TACGGTAGCAGAGACTTGGTCTCCACTTCGAGCGGAGAGA
ARMC2	1	NM_032131	CCDS5069	TACGGTAGCAGAGACTTGGTCTATCATTTGGAGACAGCATGG
CEP57L1	1	NM_001350656	CCDS87427	TACGGTAGCAGAGACTTGGTCT TTCTGGGTGAATGAGGGAAC

Other Primers		Sequence
Universal foreward primer for SIN-SIEW-CCDS clones.		ACACTGACGACATGGTTCTACAGCCAGTCCTCCGACAGAC
Foreward primer for SLIEW		ACACTGACGACATGGTTCTACACTTGGCATTCCGGTACTGTT
Reverse primer for SLIEW		TACGGTAGCAGAGACTTGGTCTGCCTTATGCAGTTGCTCTCC
Foreward Illumina adaptor primer (PE1-CS1-F)		AATGATACGGCGACCACCGAGATCTACACTGACGACATG GTTCTACA
Reverse Illumina adaptor primer (PE2-BC-CS2-R)*		CAAGCAGAAGACGGCATACGAGATGTATCGTCGTTACGG TAGCAGAGACTTGGTCT
Foreward primer for Sanger sequencing (SFFV L1)**		CTTCTGCTTCCCGAGCTCTA
Reverse primer for Sanger sequencing (IRES R1)**		AGGAACTGCTTCGTTCACGA

* Representative example, bar code (BC) sequence varies.

** Primers used for analysis of cloned CCDS inserts.

Table S11. Custom sequencing primers for amplification of GeCKO constructs.

Primer name	Primer sequence
Universal forward primer	AATGATACGGCGACCACCGAGATCTACACTCTTTCCCTACACGACGCTCTTCCGATCT NNNNNTCTTGTGGAAAGGACGAAACACCG
Reverse primer 01	CAAGCAGAAGACGGCATACGAGATAAGTAGAGGTGACTGGAGTTCAGACGTGTGCTCTTCCGATCT TTGTGGGCGATGTGCGCTCTG
Reverse primer 02	CAAGCAGAAGACGGCATACGAGATACACGATCGTGACTGGAGTTCAGACGTGTGCTCTTCCGATCT ATGTGGGCGATGTGCGCTCTG
Reverse primer 03	CAAGCAGAAGACGGCATACGAGATCGCGCGGTGTGACTGGAGTTCAGACGTGTGCTCTTCCGATCT GATGTGGGCGATGTGCGCTCTG
Reverse primer 04	CAAGCAGAAGACGGCATACGAGATCATGATCGGTGACTGGAGTTCAGACGTGTGCTCTTCCGATCT CGATGTGGGCGATGTGCGCTCTG
Reverse primer 05	CAAGCAGAAGACGGCATACGAGATCGTTACCAGTGACTGGAGTTCAGACGTGTGCTCTTCCGATCT TCGATGTGGGCGATGTGCGCTCTG
Reverse primer 06	CAAGCAGAAGACGGCATACGAGATTCCTTGGTGTGACTGGAGTTCAGACGTGTGCTCTTCCGATCT ATCGATGTGGGCGATGTGCGCTCTG
Reverse primer 07	CAAGCAGAAGACGGCATACGAGATAACGCATTGTGACTGGAGTTCAGACGTGTGCTCTTCCGATCT GATCGATGTGGGCGATGTGCGCTCTG
Reverse primer 08	CAAGCAGAAGACGGCATACGAGATACAGGTATGTGACTGGAGTTCAGACGTGTGCTCTTCCGATCT CGATCGATGTGGGCGATGTGCGCTCTG
Reverse primer 09	CAAGCAGAAGACGGCATACGAGATAGGTAAGGGTGACTGGAGTTCAGACGTGTGCTCTTCCGATCT ACGATCGATGTGGGCGATGTGCGCTCTG
Reverse primer 10	CAAGCAGAAGACGGCATACGAGATAACAATGGGTGACTGGAGTTCAGACGTGTGCTCTTCCGATCT TGTGGGCGATGTGCGCTCTG
Reverse primer 11	CAAGCAGAAGACGGCATACGAGATACTGTATCGTGACTGGAGTTCAGACGTGTGCTCTTCCGATCT ATGTGGGCGATGTGCGCTCTG
Reverse primer 12	CAAGCAGAAGACGGCATACGAGATAGGTCGCAGTGACTGGAGTTCAGACGTGTGCTCTTCCGATCT GATGTGGGCGATGTGCGCTCTG

**Table S12. Primers used for RT-PCR of candidate 6q TS genes**			
**Primer name**	**Sequence**	**product size (bp)**	**REFseq ID**
FOXO3 1F	CACGTCTTCAGGTCCTCCTGT	137	NM_001455.4
FOXO3 1R	GGGAAGCACCAAAGAAGAGAGAA		
FOXO3 2F	TCTTCAGGTCCTCCTGTTCCTG	132	
FOXO3 2R	GGAAGCACCAAAGAAGAGAGAAG		
PRDM1 F1	CAGACAAAGTGCTGCCGTGA	195	NM_001198.4
PRDM1 R1	TACCCAGTCCACATTCTCCCC		
PRDM1 F2	GTGTTGCGGAGAGGCAAGAG	159	
PRDM1 R2	TCTACCCAGTCCACATTCTCC		
POU3F2 F1	GAAAAGGATGACCCCTCCCG	78	NM_005604.4
POU3F2 R1	TGTGGTGGAGTGTCCCTACT		
POU3F2 F2	AAATGCGCGGCTCCTTTAAC	86	
POU3F2 R2	GTGAGCAGGCTGTAGTGGTT		
PRDM13 F1	GTCTGCTTCACAGACGACCA	161	NM_021620.4
PRDM13 R1	CATTGGGTCCCTCTTCCCAC		
PRDM13 F2	ACCTGGAGCGACATGTCAAG	146	
PRDM13 R2	GTCTGTGAAGCAGACGTCCA		
SIM1 F1	CTCGTCCCAAAGCTTGCATC	151	NM_005068.3
SIM1 R1	TCTCATGCCTACCGTCCAAC		
SIM1 F2	ATCCTCCTCTTTCTGCCACG	136	
SIM1 R2	GTTCTCATGCCTACCGTCCA		
FAXC F1	GCAGCATGCACTGGGGG	151	NM_032511.4
FAXC R1	TCCTGCAAAGGGAAAGCGAT		
FAXC F2	GCGCAGCATGCACTGG	154	
FAXC R2	ATCCTGCAAAGGGAAAGCGA		

Table S13. Primers used for ddPCR			
**Primer name**	**Sequence**	**product size (bp)**	**REFseq ID**
IRF4 F1	CCATGACAACGCCTTACCCT	123	NM_002460.4
IRF4 R1	ATTGGTACGGGATTTCCGGG		
CXCR4 F1	GCAGCAGGTAGCAAAGTGAC	78	NM_003467.3
CXCR4 R1	CCATGGTAACCGCTGGTTCT		
SPIB F1	GCTGCAAGCATTCCAGCTAC	104	NM_003121.5
SPIB R1	GTCGAAGGCTTCATAGGGGG		
GADD45A F2	ACGATCACTGTCGGGGTGTA	106	NM_001924.4
GADD45A R2	CCACATCTCTGTCGTCGTCC		
TXNIP F1	GGAGTGCTTGTGGAGATCGG	88	NM_006472.6
TXNIP R1	ACGCCGCTGGTTACACTAAG		
RP2 F2	GACGGTAGCAGGACAACAGT	148	NM_006915.3
RP2 R2	ATTCCGGAAAAACACGCTGC		
TBP F1	GCAAGGGTTTCTGGTTTGCC	156	NM_003194.5
TBP R1	GGGTCAGTCCAGTGCCATAA		

Supplementary Figures

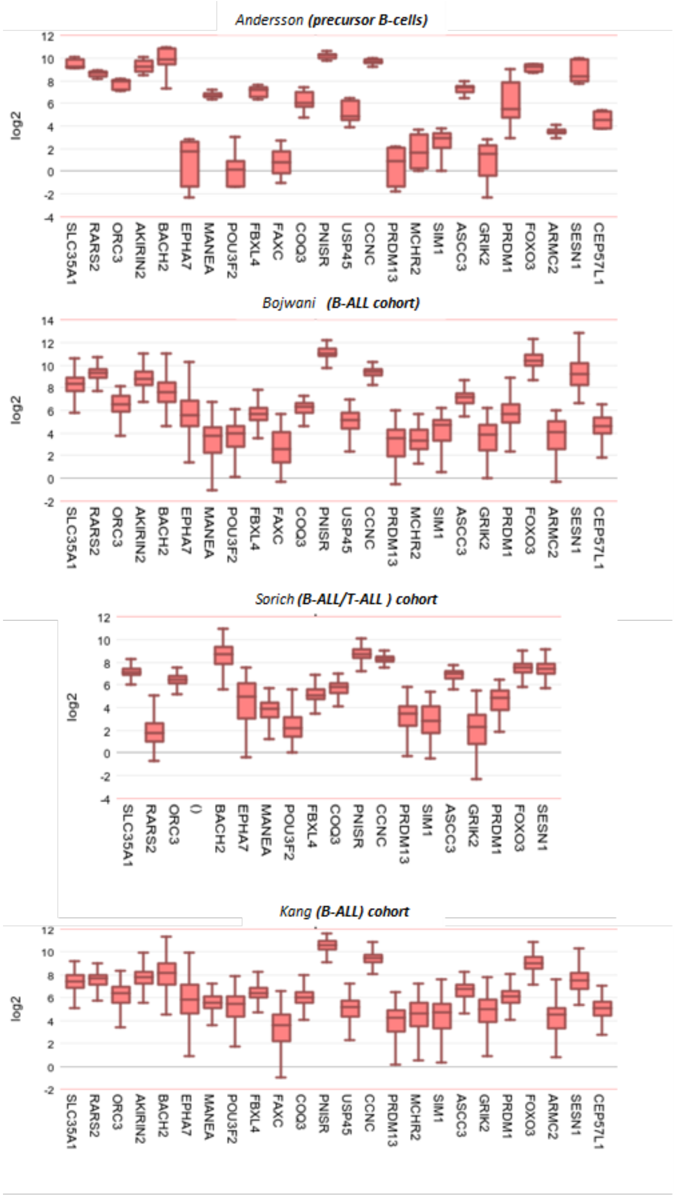

Supplementary Figure 1

A

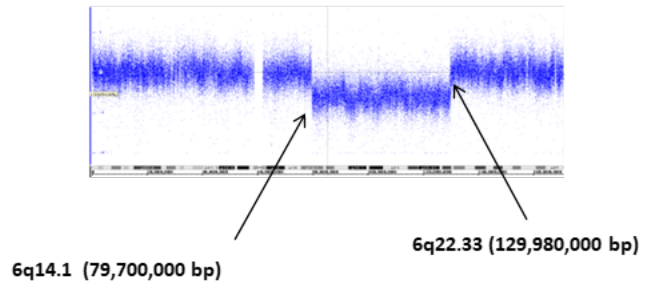

B

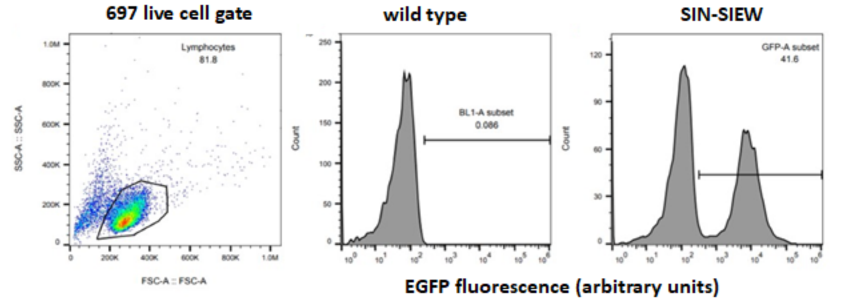

Supplementary figure 2

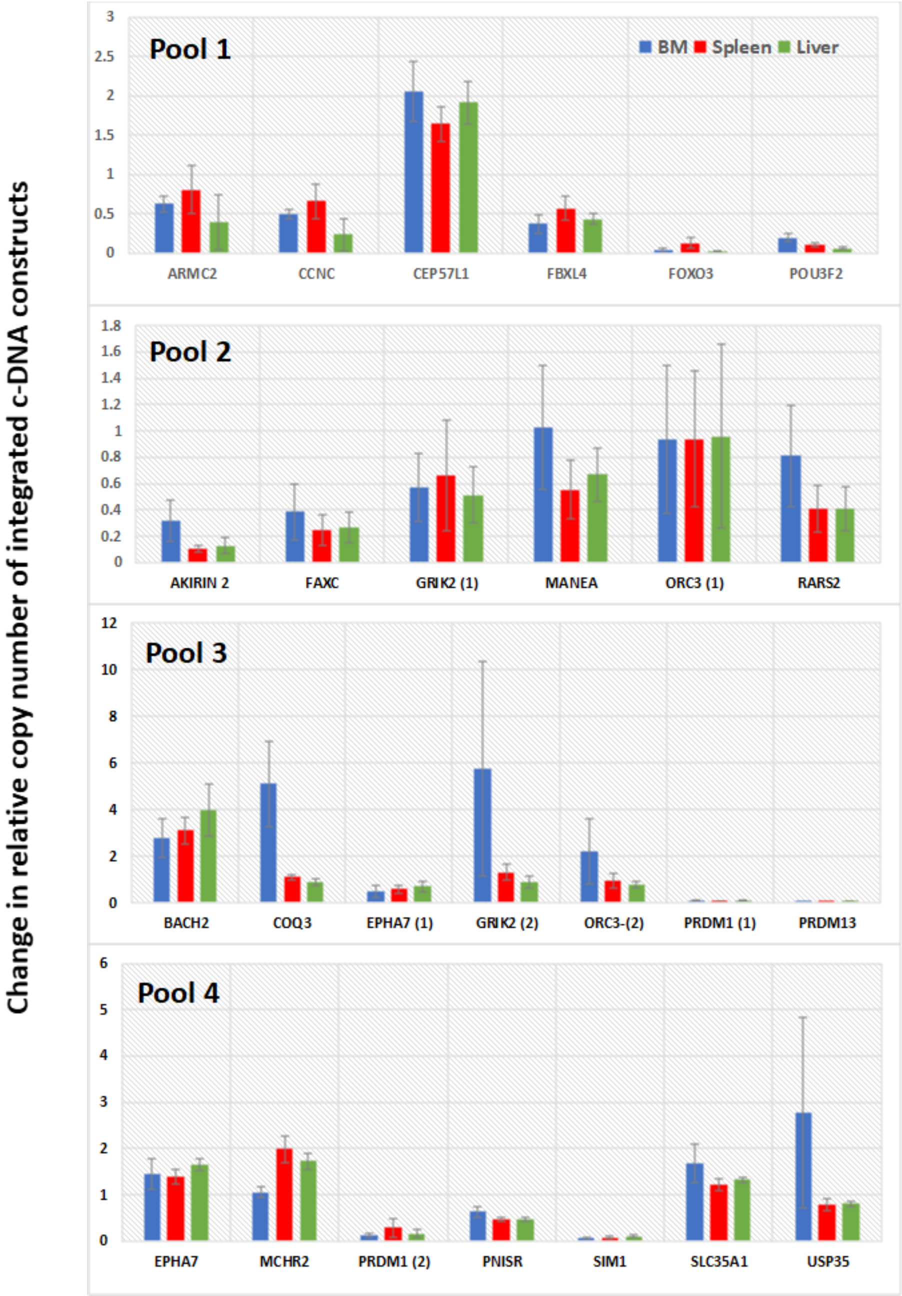

Supplementary Figure 3

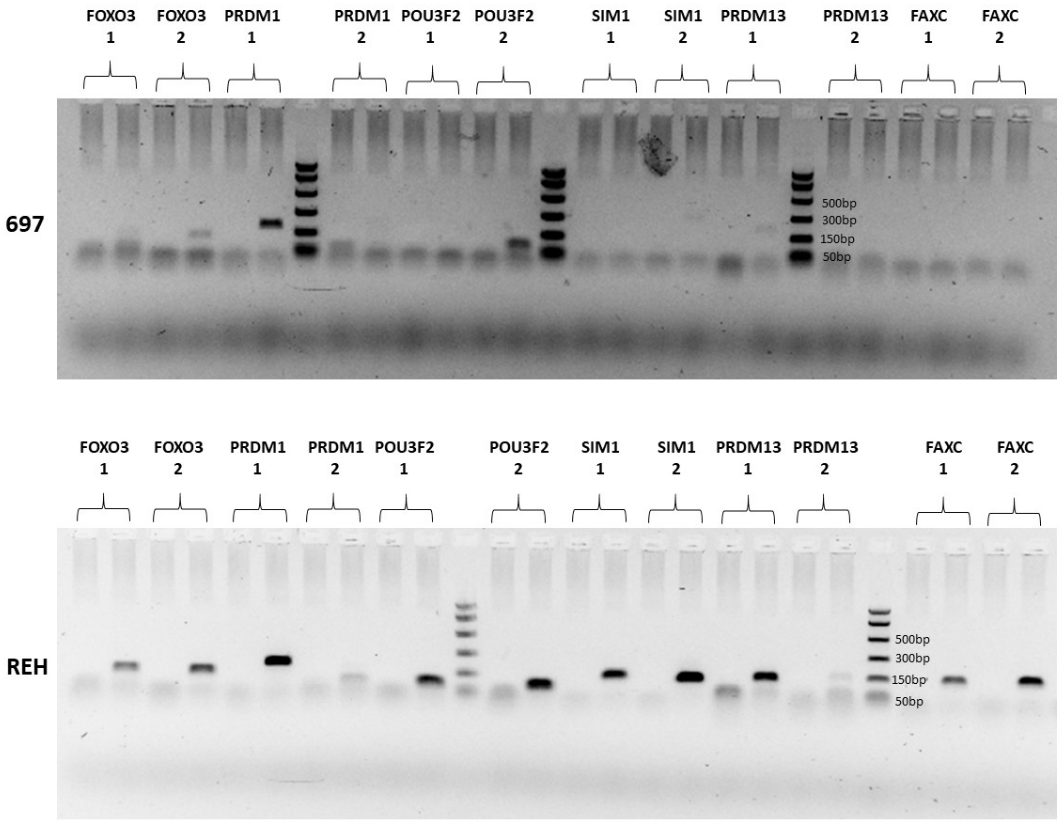

Supplementary figure 4

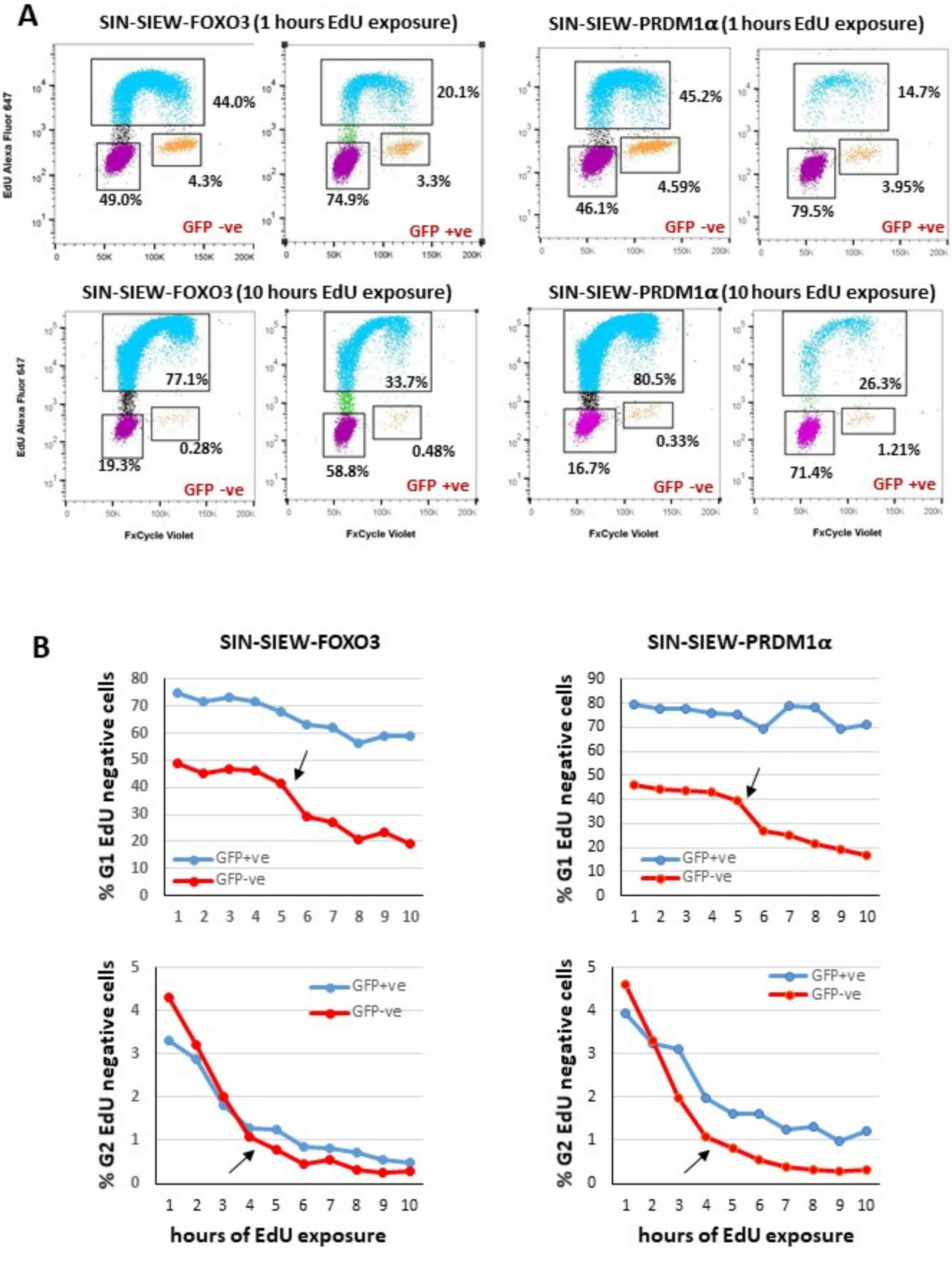

Supplementary Figure 5

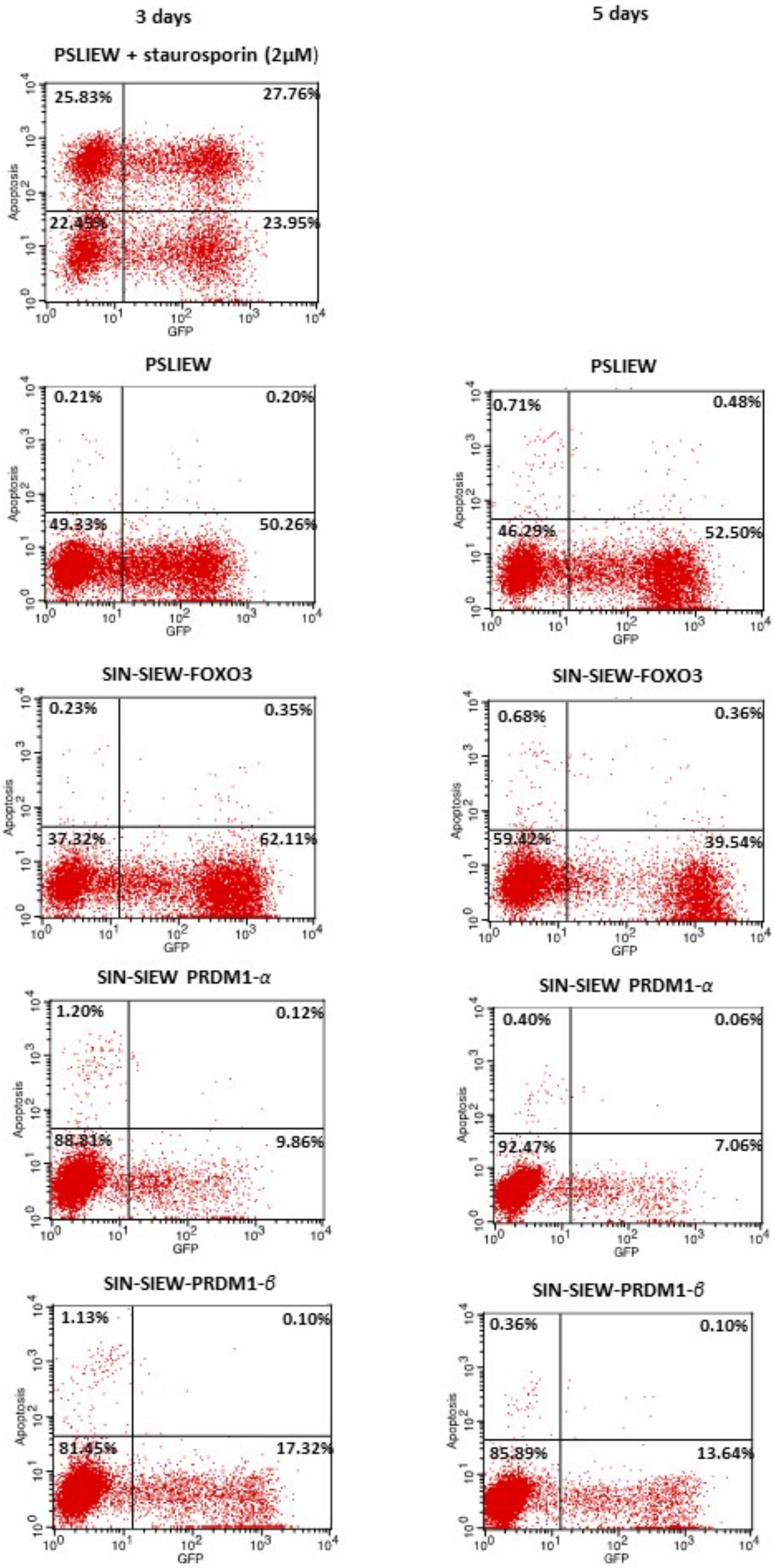

Supplementary Figure 6

Supplementary figure legends.

Figure S1. Relative expression of genes positioned within regions of focal deletion of 6q in ALL. Comparative expression levels of all genes associated with recurrent focal deletions of 6q are shown for normal human mixed pro-B, pre-B and immature B cells (precursor B cells) and in three published ALL cohorts. Data sets were generated from micro-array experiments archived under GEO accession numbers; Andersson (GSE19599), Bojwani (GSE7440), Kang (GSE11877) and Sorich (GSE10255) and accessed and analysed in the R2 genomics and visualisation platform(https://hgserver1.amc.nl/cgi-bin/r2/main.cgi). No gene shows clear evidence for repressed expression in ALL when compared with precursor B-cells.

Figure S2. **Characterisation of 697**. **A**. SNP6.0 array copy number profile of chromosome 6 from B-ALL cell line 697. Normal copy number is represented by the horizontal line. Breakpoints for a monoallelic deletion of the long arm, between 79.70 and 129.98 Mb, are indicated by arrows. **B**. Example of flow cytometric analysis of 697 cells, showing the live cell gate and a distinct peak of EGFP positive cells 72 hours following transduction with SIN-SIEW.

Figure S3. ***In-vivo* clone tracking assays**. 697 cells transduced with pools of lentivirus consisting of pSLIEW and SIN-SIEW expressing cDNAs for the genes indicated were transplanted into femurs of NSG mice. Samples were isolated from bone marrow, spleen and liver after mice developed leukaemia. Histograms show the change in mean construct copy numbers, relative to SIN-SIEW, between pre-transplant and post-mortem samples. Numbers 1 and 2 distinguish between different CCDS encoded by a single gene. Error bars indicate standard error of the mean (SEM).

Figure S4. Expression of candidate 6qTS genes in 697 and REH B-ALL cells. PCR reactions were performed with two primers pairs (1 and 2) and NT control (left well) or cDNA (right well) synthesised from 697 RNA (top gel) or REH RNA (bottom gel). Visible PCR products of expected size (table S12) were amplified by FOXO3-1 and -2, PRDM1-1, POU3F2-2, and PRDM13-1 primers from 697 cDNA. From REH cDNA, expected products were amplified by all primer pairs, except PRDM1-2. No gene specific products amplified from the NT controls.

Figure S5. Effects of expression of FOXO3 and PRDM1 on cell cycle dynamics. Cells were exposed to EdU for between 1 and 10 hours, four days after transduction with SIN-SIEW-FOXO3 or SIN-SIEW-PRDM1α. EGFP negative cells act as internal controls for comparison with EGFP positive cells expressing FOXO3 or PRDM1. Percentages of EdU positive and negative stained cells in EGFP expressing and non-expressing populations were compared after different lengths of EdU exposure. **A.** Examples of cells exposed to EdU for 1 and 10 hours. Gated cells are; G1 EdU negative (purple) G2 EdU negative (orange) and EdU positive (cyan), percentage of the total is indicated for each gate. In both FOXO3 and PRDM1 transduced cultures, depletion of EdU G1 and G2 EdU negative populations is attenuated in EGFP positive compared with EGFP negative cells. **B**. Plots showing changes in the percentage of cells in EdU negative G1 and G2 gates over a 10-hour time course for EGFP positive and negative populations. In both SIN-SIEW-FOXO3 and SIN-SIEW-PRDM1

transduced cultures, EGFP negative cells show a sharp decline in the percentage of G2 EdU negative cells between 1 and 4 hours, indicative of the duration of G2. Consistent with this observation, an inflection in the rate of G1 cell loss occurred between 5 and 6 hours indicating G2-G1 transition of the first EdU labelled cells (indicated by arrows). In EGFP expressing cells, phases of cell the cell cycle are less clearly demarcated and proportions of EdU negative cells are relatively higher at the end of the time course, consistent with cell cycle delay or block at both the G1 and G2 stages. Comparison between the two constructs suggests that G2 passage is more potently inhibited by PRDM1 than by FOXO3 expression.

Figure S6. **FOXO3 and PRDM1 do not induce apoptosis.** Flow cytometry plots of levels of surface Annexin 5 staining (marking apoptotic cells) in EGFP positive and negative populations at three days and five days following transduction of 697 cells with SIN-SIEW constructs expressing *FOXO3*, *PRDM1-*α and *PRDM1-*β. A four-hour exposure to staurosporin was used as a positive control for the apoptosis assay.
